# Assessing causality between inflammatory bowel diseases with frailty index and sarcopenia: a bidirectional Mendelian randomization study

**DOI:** 10.1186/s40001-023-01614-5

**Published:** 2024-01-05

**Authors:** Peng Wang, Wei Tao, Zhiqiang Zhang, Cong Xu, Yuan Qiu, Weidong Xiao

**Affiliations:** Department of General Surgery, Xinqiao Hospital, Army Medical University, No. 183 Xinqiao Road, Chongqing, 400037 China

**Keywords:** Mendelian randomization, Inflammatory bowel disease, Frailty, Sarcopenia

## Abstract

**Background:**

Previous studies have found that frailty and sarcopenia are commonly diagnosed in inflammatory bowel disease (IBD) patients, indicating an association between these conditions. Nonetheless, the cause‒effect connection between IBD, frailty, and sarcopenia remains unclear.

**Methods:**

We sourced the genetic variants for the exposures and outcomes from publicly accessible, extensive genome-wide association studies (GWAS). Specifically, we obtained IBD data from the International IBD Genetics Consortium, frailty index (FI) data from the United Kingdom Biobank and Swedish TwinGene, and sarcopenia data from a recent GWAS meta-analysis. Five methods, including inverse variance weighted (IVW), simple mode, MR-Egger, weighted mode, and the weighted median, were used to proceed with MR estimates. We also performed heterogeneity and horizontal pleiotropy tests.

**Results:**

Our results indicated a positive causal relationship between ulcerative colitis (UC) (IVW: *β* = 0.014, 95% CI, 0.006 to 0.021, *p* = 0.001) and Crohn's disease (CD) (IVW: *β* = 0.012; 95% CI, 0.006 to 0.018, *p* = 2e−04) with the FI. However, we uncovered no proof of a cause-and-effect relationship between UC (IVW: *β* = 0.001, 95% CI, −0.015 to 0.017, *p* = 0.344) or CD (IVW: *β* = 0.003, 95% CI, −0.009 to 0.015, *p* = 0.214) and sarcopenia. Additionally, in the inverse order, we also discovered no cause-and-effect connection between FI or sarcopenia on UC or CD in this study.

**Conclusion:**

The MR analysis showed a positive causal association between IBD and FI, indicating that IBD patients may exhibit aging-related characteristics. Therefore, frailty assessments should be conducted as early as possible in IBD patients.

**Supplementary Information:**

The online version contains supplementary material available at 10.1186/s40001-023-01614-5.

## Introduction

Inflammatory bowel disorder (IBD), encompassing both Crohn's disease (CD) and ulcerative colitis (UC), represents a chronic and recurring inflammatory situation chiefly impacting the digestive system. There are nearly 7 million cases worldwide [1]. While the most common onset of IBD is typically at a relatively young age, the occurrence and commonness of this ailment persistently escalates in conjunction with a progressively aging populace [2]. Due to more effective drug and endoscopic treatments, the progression of IBD has been mitigated, but the prevalence of older individuals diagnosed with IBD is also increasing [3]. IBD often leads to repeated bowel damage and impaired nutrient absorption in the gastrointestinal tract [4]. This malabsorption, along with advancing age, can escalate the risk of disease progression and increase the likelihood of complications and higher medical costs in IBD patients [4, 5]. Furthermore, Nakov R et al. reported that relatively young IBD patients were associated with more frequent episodes of inflammation, which could exacerbate the adverse outcomes of IBD [6].

Frailty and sarcopenia are disease states associated with malnutrition, advanced age, and chronic inflammation [7, 8]. Although frailty and sarcopenia are distinct concepts, it is important to understand that sarcopenia can be viewed as a physical manifestation of frailty [9]. Frailty is characterized by increased vulnerability due to age-related deterioration across multiple physiological systems [10]. In the context of IBD, prior studies have linked the presence of frailty to a range of adverse outcomes, such as an increased risk of serious infections, unfavorable postoperative results [11]. Similarly, sarcopenia is identified as a syndrome marked by a progressive decrease in skeletal muscle mass, leading to diminished muscle strength and functional impairment [12]. A previous study found that sarcopenia was common among IBD patients and was linked with an elevated risk of unfavorable surgical outcomes and severe clinical outcomes [13].

Recent years have seen increased interest in studies connecting IBD with frailty or sarcopenia. Kochar BD et al. discovered that 6% of IBD patients could be diagnosed with frailty, which might independently predict mortality [8]. Frailty is a dynamic state linked to dysregulated immune and endocrine systems and chronic inflammation. Treating IBD patients with frailty using effective anti-inflammatory therapy could improve their condition [14]. Multiple studies support that frailty is associated with chronic inflammation. Ferrucci L et al. reported that elevated serum interleukin (IL)-6 levels were connected to reduced muscle strength, a phenotype of frailty [15]. However, Renier AP et al. found no association between IL-6 and frailty [16]. Thus, the precise causal relationship between frailty and IBD remains to be determined. Sarcopenia was identified as a negative factor in clinical outcomes for IBD patients [17]. Chronic inflammation, damaged mucosa, dysregulated adipose tissue, and malabsorption might be the mechanisms driving sarcopenia in IBD [18]. Consequently, individuals diagnosed with inflammatory bowel disorder face a heightened likelihood of developing sarcopenia. However, many studies on IBD have primarily focused on evaluating muscle mass parameters when assessing sarcopenia. This narrow approach might lead to higher heterogeneity and obscure the relationship between sarcopenia and IBD. Despite multiple observational investigations and randomized controlled experiments related to IBD with frailty and sarcopenia, the direct cause–effect connection between them continues to be ambiguous.

Mendelian randomization (MR) is a technique that employs genetic variations as instrumental variables (IVs) to explore the fundamental cause–effect relationship between an exposure and a result. These genetic variants, randomly assigned during meiosis and fertilization, are generally unaffected by self-selected behaviors. Their inherent independence, established well before the onset of disease, helps to mitigate concerns about confounding factors and reverse causality [19].

In this bidirectional MR study, the IVs for IBD, frailty, and sarcopenia were derived from extensive genome-wide association studies (GWAS) using nonoverlapping samples, offering reliable summary statistics. The main aim of this research was to apply a bidirectional MR analysis to investigate the causality between IBD and both frailty and sarcopenia.

## Methods

### Study design overview

**Fig. 1:**
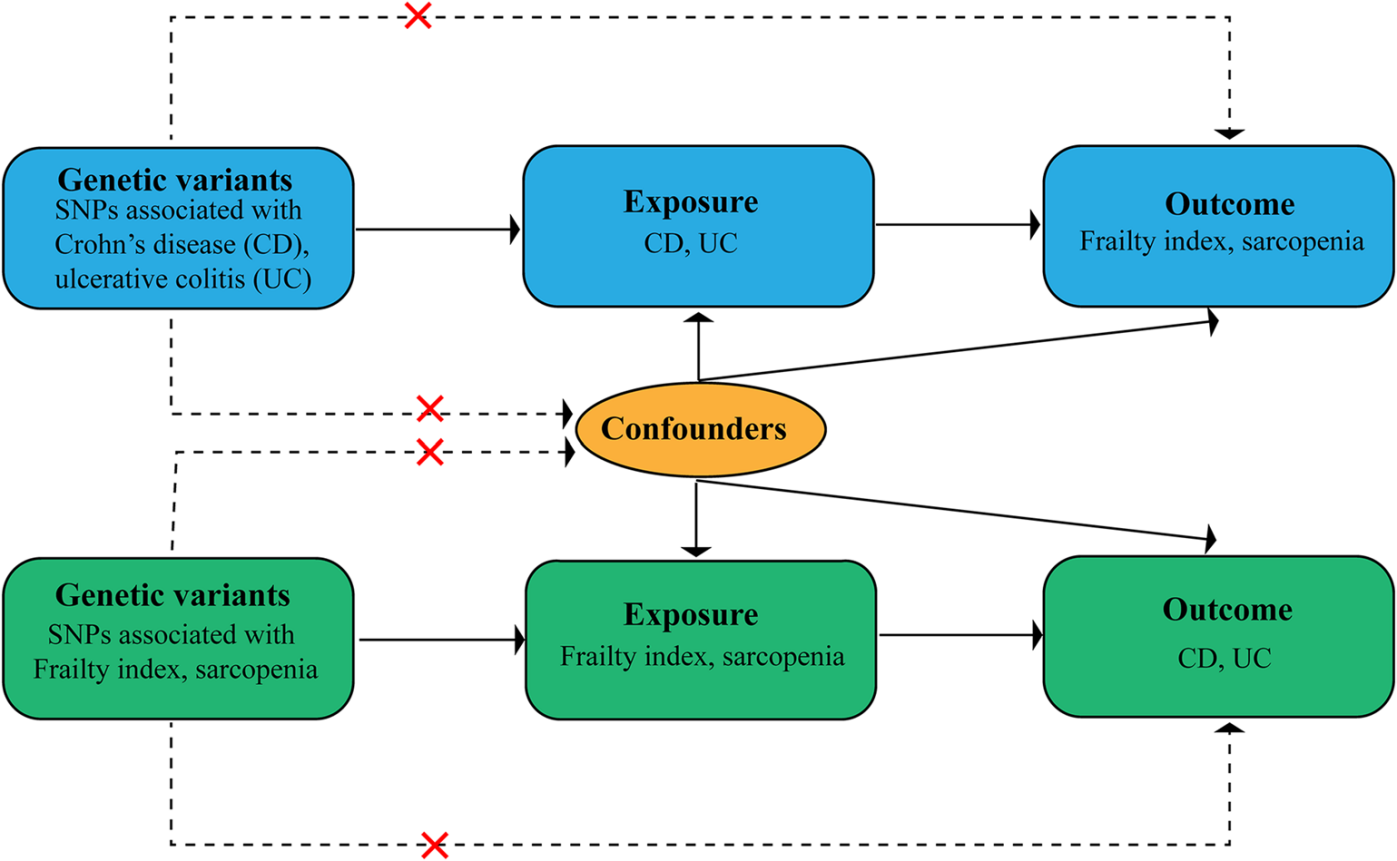
The methodology of implementing a bidirectional Mendelian randomization (MR) examination. The sign " × " suggests that genomic changes are disconnected from confounding elements or do not directly affect the outcome. Rather, they wield their impact via the exposure pathway. A persistent line is indicative of a substantial link, while a discontinuous line shows no interrelation between the variables. SNP: single nucleotide polymorphism; UC, ulcerative colitis; CD, Crohn’s disease

Figure 1 offers a comprehensive overview of our bidirectional MR study design. In brief, this study estimated the causal effects of UC and CD on the frailty index (FI) and sarcopenia. Subsequently, we investigated the cause-and-effect impacts of FI and sarcopenia on UC and CD. For genetic variants to be considered IVs, they must satisfy three stringent assumptions: first, the genetic variations are significantly linked with the exposure variable; second, these genetic variations are not connected with any confounding elements. Last, the genetic variants do not directly affect the outcome, but rather, they influence it through the exposure pathway [20].

### IBD, frailty index and sarcopenia genome-wide association study (GWAS) summary statistics

To guarantee the sturdiness and dependability of the cause-and-effect connection between IBD, FI, and sarcopenia, we specifically utilized the most extensive GWAS currently available for IBD, which encompassed both UC and CD [21]. Complete summary statistics for the IBD GWAS (IDs: ieu-a-32 and ieu-a-30) were available for download from the IEU Open GWAS Project website at https://gwas.mrcieu.ac.uk. We acquired comprehensive aggregated data from the European GWAS for UC with a case count of 6968 and 20,464 controls, along with CD presenting 5956 cases and 14,927 controls. These statistics were sourced from the globally recognized authority on the subject, the International Inflammatory Bowel Disease Genetics Consortium.

We retrieved summary statistics for frailty, assessed using the FI phenotype, from a contemporary comprehensive review of GWAS undertaken in prominent research institutions such as the United Kingdom (UK) Biobank and the Swedish TwinGene cohorts. This encompassed a considerable number of 175,226 individuals, all of whom share a European lineage [22].

The summary statistics for sarcopenia in our study were acquired from the GWAS catalog database. These data, used in our study, were extracted from a comprehensive GWAS meta-analysis conducted by Jones et al. [23]. The repositories of information for both the exposure and the resultant outcomes are comprehensively encapsulated in Table 1.

**Table 1:** All the GWAS summary data were used in this Mendelian randomization study

Phenotype	Consortium	Year	Cases (*N*)	Sample size (*N*)	GEAS ID
UC	IIBDGC	2015	6968	27,432	ieu-a-32
CD	IIBDGC	2015	5956	20,883	ieu-a-30
Frailty index	NA	2021	NA	175,226	ebi-a-GCST90020053
sarcopenia	NA	2021	48596	256,523	ebi-a-GCST90007526

*UC* ulcerative colitis, *CD* Crohn’s disease

### Frailty index (FI)

The FI is a continuous, wide-ranging metric that amalgamates over 40 elements spanning numerous physiological and psychological health areas. This assessment method is adept at encapsulating age-related health inadequacies, thus offering a comprehensive view of an individual's aging health status [24]. It has been widely validated and recognized for its robust predictive capability concerning various unfavorable health outcomes, making it a reliable indicator of overall health. Additionally, the FI is particularly suitable for assessing frailty in overall health level compared to alternative measures [25].

### Genetic instrumental variable selection

Within the structure of MR, we handpicked particular single nucleotide polymorphisms (SNPs) to serve as instrumental variables IVs based on the foundational premise of MR. The selection of IVs was guided by three assumptions (Fig. 1): (1) a significant association exists between the IVs and the exposure; (2) there are no pleiotropic correlations between the IVs and any known confounding factors; and (3) the IVs have no potential effect on the outcome, excluding any potential mediating effects via the exposure variable associated with the IVs [26]. The IVs utilized in our research were extracted from previously reported GWAS using the clumping function within the two-sample MR package. All IVs and exposure traits, except for FI and sarcopenia (*p* < 5 × 10^–7^), demonstrated significant independent associations (*p* < 5 × 10^–8^), with bidirectional linkage disequilibrium (LD) *r*^2^ < 0.01 [27]. Following established guidelines, we identified a set of independent SNPs highly associated with exposure variables, which were subsequently matched in the outcome database. We used the harmonized data function provided by the two-sample MR package to achieve harmonization and to match the effect allele of each SNP with the corresponding allele of the exposure variable, ensuring consistent alignment for subsequent analyses.

### Assessment of horizontal pleiotropy and heterogeneity

In the context of the inverse variance weighted (IVW) study, it is crucial to consider the potential impact of pleiotropy on causal estimates and findings [27]. To assess the suitability of the selected SNPs as IVs, we used the two-sample MR package to test for pleiotropy. By conducting this test, we evaluated whether significant pleiotropy was present (*p* > 0.05) and determined the appropriateness of using the IVs. Moreover, we utilized Cochran's Q test as a statistical tool to evaluate the level of inconsistency or diversity present among the chosen IVs. If the heterogeneity was found to be negligible, we employed the fixed-effects model. However, if significant heterogeneity was observed, we proceeded with the use of the random-effects IVW approach for the analysis.

### Analysis of MR

MR harnesses the power of genetic IVs to scrutinize and evaluate the cause-and-effect relationship between the exposure variable and the resultant outcome. In our study, we initially computed the Wald ratio for each IV by dividing outcome data by its corresponding exposure data. Next, we employed the IVW approach to estimate the relationship between the exposures and the outcomes. The IVW analysis utilized the inverse variance technique to assign weight to the Wald ratio of each SNP, considering the meta-analysis influence through either random or fixed-effects estimation. If the significance level derived from Cochran's Q test was below the 0.05 threshold, random-effect models were implemented to account for the observed variability; in contrast, if this threshold was not reached, fixed-effect models were adopted to maintain consistent effect sizes across the different studies. To supplement the findings obtained through the IVW analysis, we also employed additional approaches, such as MR-Egger, weighted mode, simple mode, and the weighted median approach. These methods provided supplementary insights and enhanced the robustness of our results.

### Testing instrument strength

The F statistic acts as an indicator of the robustness of the instrument, encapsulating the relationship among the proportion of variability in the phenotype accounted for by the genetic variants (*R*^2^), the total number of observations (*N*), and the quantity of instruments (*k*). It can be calculated using the formula *F* = *R*^2^(*N*−*k*−1)/*k* (1−*R*^2^) [28]. To compute the *R*^2^ for each instrument *i*, we utilize the approximation *Ri*^2^ = 2 × EAFi × (1−EAFi) × βi^2^, where EAFi symbolizes the frequency of the impact allele, and βi signifies the calculated influence of the genetic variant on the exposure variable [29]. In MR analysis, an *F* statistic of ≥ 10 is typically considered indicative of a relatively low risk of weak instrument bias [30]. This threshold helps ensure the robustness of the MR analysis by indicating sufficient instrument strength to draw reliable causal inferences.

### Sensitivity analysis

To ascertain the strength and reliability of the MR causal effect estimate, we performed a series of sensitivity analyses. Initially, we utilized the MR-Egger intercept method to evaluate the potential existence of pleiotropy among the chosen SNPs. If the intercept term demonstrated statistical significance (*p* < 0.05), it suggested the possible occurrence of pleiotropic effects. Conversely, no horizontal pleiotropy among the IVs was found (*p* > 0.05). Second, we harnessed the Mendelian randomization pleiotropy residual sum and outlier (MR-PRESSO) test as a tool to pinpoint any anomalies in the data and to rectify them to procure a dependable estimate. A subsequent sensitivity analysis was carried out to evaluate the influence of the MR-PRESSO adjustment on the IVW causal effect. Additionally, a "leave-one-out" method, which involved sequentially removing each individual SNP, was performed to enhance the robustness of the findings.

### Reported findings and software

The findings from the MR analysis were presented as estimated values. For binary variables, we employed odds ratios (ORs) accompanied by 95% confidence intervals (CIs), while for continuous variables, beta values (*β*) with 95% CIs were employed. These estimated values were consistently reported throughout the analysis. Accurate and reliable results were obtained by conducting the statistical analysis using the packages of two-sample MR and MR-PRESSO in R version 4.2.0.

## Results

### A two-sample MR analysis for the potential causal relationship between UC and CD on FI and sarcopenia

**Fig. 2:**
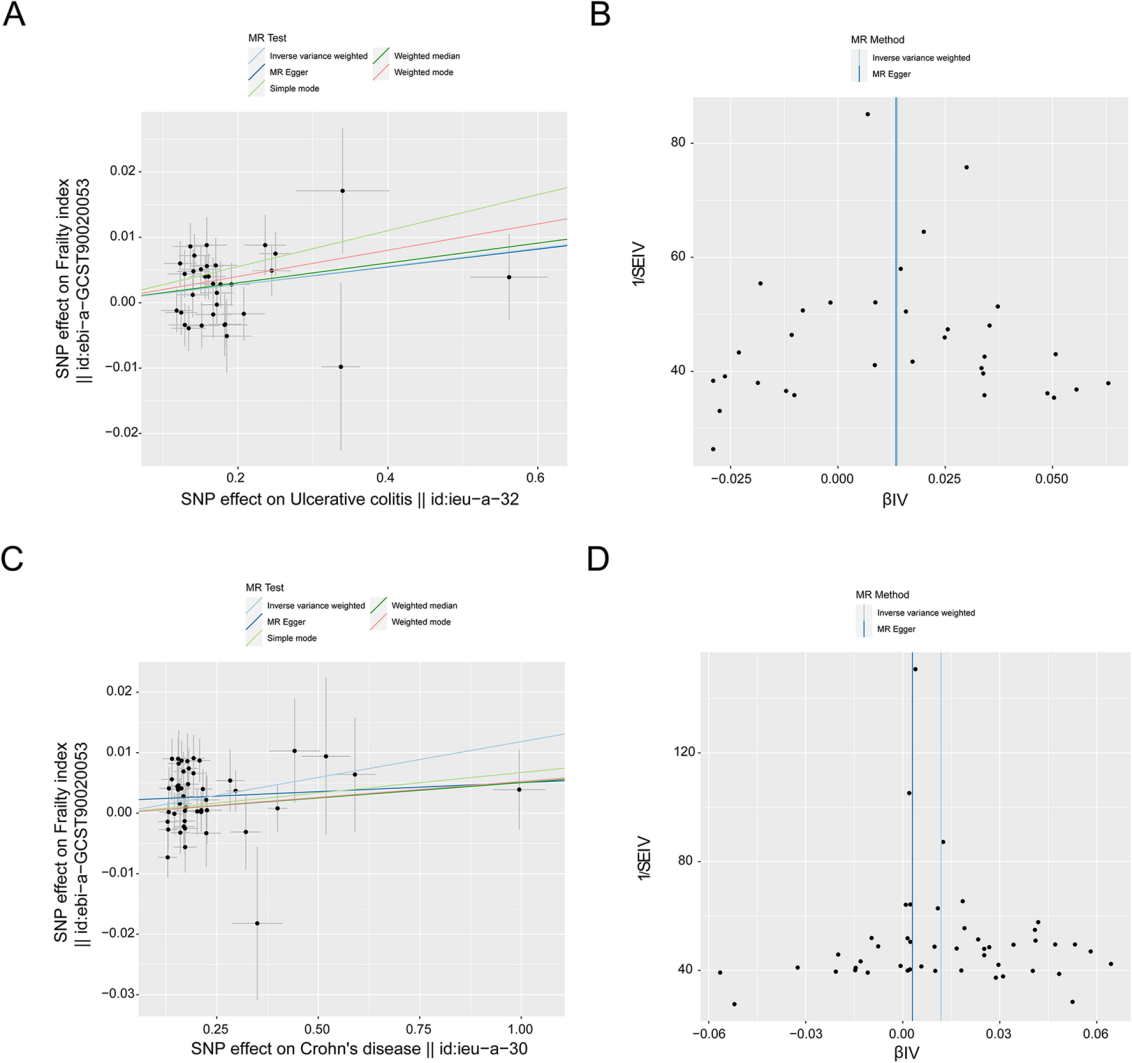
Point diagrams and a funnel illustration were used to portray the causative impact of UC and CD on the frailty index. Research focusing on the influence of UC (**A**, **B**) on the frailty index and the impact of CD (**C**, **D**) on the frailty index was performed. These studies implemented conventional IVW, simple mode, MR Egger, weighted median and weighted mode methods. The inclination of each line served to compute the MR influence per approach. A funnel chart was adopted for the analysis of variance in the data. The azure line characterizes the IVW prediction, while the deep blue line exhibits the MR-Egger prediction

In this research, we carried out a two-sample MR, which used genetic variants associated with UC and CD with FI and sarcopenia originating from a contemporary GWAS of European ancestry. The MR analysis showed a positive causality between UC and FI, and the occurrence of UC was positively correlated with an elevated FI (IVW: *β* = 0.014, 95% CIs, 0.006–0.021, *p* = 0.001; MR‒Egger: *β* = 0.013, 95% CIs, −0.013–0.041, *p* = 0.333; weighted median: *β* = 0.015, 95% CIs, 0.004–0.026, *p* = 0.007; simple mode: *β* = 0.028, 95% CIs, 0.004–0.051, *p* = 0.032; weighted mode: *β* = 0.020, 95% CIs, 0.002–0.038, *p* = 0.032) (Fig. 2A, Table 2, in Additional file 1: Fig. S1A). No heterogeneity was detected in certain results (IVW: Q-value = 39.764, *p* = 0.163; MR-Egger: Q-value = 39.763, *p* = 0.134). Based on the MR-PRESSO analysis, no potential outliers were detected (*p* = 0.186). The employment of the MR-Egger intercept approach failed to yield significant evidence of directional pleiotropy among the selected IVs (*p* = 0.979). The funnel plot showed that there was no bias in the study (Fig. 2B, Table 2). The leave-one-out evaluation suggested that none of the individual SNPs exerted a statistically significant impact on the bias of the overall causal estimates (Additional file 1: Fig. S1B). All selected SNPs included or excluded in MR analysis for replication are presented in Additional file 2: Table S1.

**Table 2:** MR results for the relationship between UC, CD and frailty index

Method	Number of SNPs	MR analysis			MR-Egger intercept *p*	Heterogeneity *p*
		Beta	95% CI	*p*		
*UC* → *FI*						
MR Egger	33	0.013	−0.013 to 0.041	0.333	0.979	0.134
Weighted median	33	0.015	0.004 to 0.026	0.007		
IVW	33	0.014	0.006 to 0.021	0.001		0.163
Simple mode	33	0.028	0.004 to 0.051	0.032		
Weighted mode	33	0.020	0.002 to 0.038	0.032		
*CD* → *FI*						
MR Egger	47	0.003	−0.011 to 0.017	0.676	0.170	0.070
Weighted median	47	0.005	−0.004 to 0.014	0.250		
IVW	47	0.012	0.006 to 0.018	2e−04		0.054
Simple mode	47	0.007	−0.011 to 0.024	0.448		
Weighted mode	47	0.005	−0.004 to 0.015	0.286		

*UC* ulcerative colitis, *CD* Crohn’s disease, *FI* frailty index, *IVW* inverse variance weight, *MR* Mendelian randomization, *SNP* single nucleotide polymorphism

Similarly, MR analysis provided robust proof of a beneficial causal link between CD and FI, with the occurrence of CD positively correlated with an elevated FI (IVW: *β* = 0.012; 95% CIs, 0.006 −0.018, *p* = 2e−04; MR-Egger: *β* = 0.003, 95% CIs, −0.01–0.017, *p* = 0.676; weighted median: *β* = 0.005, 95% CIs, −0.004–0.014, *p* = 0.250; simple mode: *β* = 0.007, 95% CIs, −0.011–0.024, *p* = 0.448; weighted mode: *β* = 0.005, 95% CIs, −0.004–0.015, *p* = 0.286) (Fig. 2C, Table 2, Additional file 1: Fig. S2A). No heterogeneity was detected in certain results (MR-Egger: Q-value = 59.795, *p* = 0.069; IVW: Q-value = 62.381, *p* = 0.054). No potential outliers were detected by MR-PRESSO (*p* = 1.000), and the use of the MR-Egger intercept method did not show any substantial indications of directional pleiotropy among the selected IVs (*p* = 0.170). The funnel plot indicated that the study was not biased (Fig. 2D, Table 2). The leave-one-out assessment demonstrated that none of the individual SNPs had a statistically significant impact on the bias of the overall causal estimates (Additional file 1: Fig. S2B). All selected SNPs included or excluded in the MR analysis for replication are presented in Additional file 2: Table S2.

**Fig. 3:**
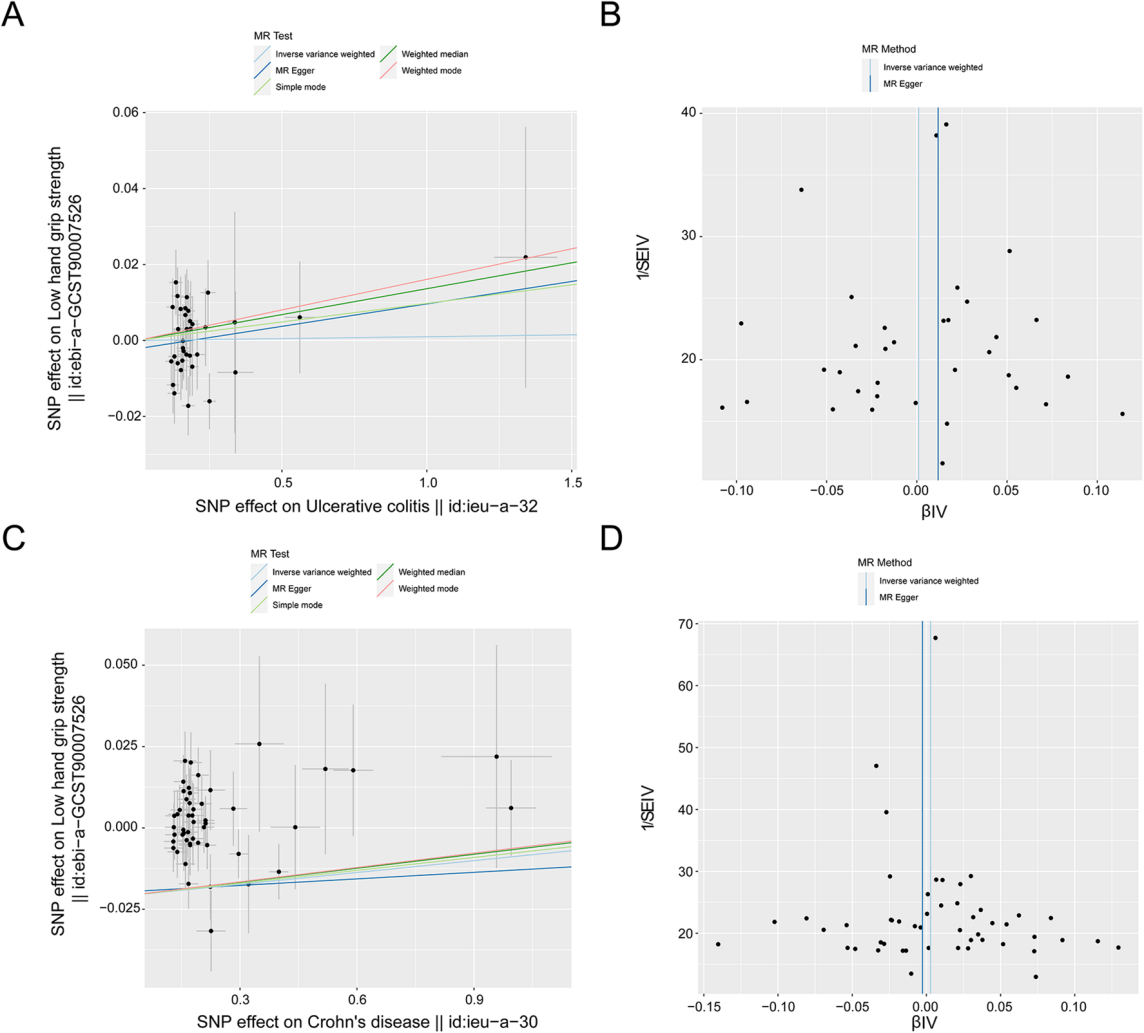
Point diagrams and a funnel chart were utilized to demonstrate the causative relationship of UC and CD with sarcopenia. This encompassed studies on the impact of UC on sarcopenia (**A**, **B**) and the influence of CD on sarcopenia (**C**, **D**). Analyses were performed employing traditional IVW, simple mode, MR Egger, weighted median, and weighted mode methods. The slope of each line represents the estimated MR impact per technique. A funnel chart was used to assess the dispersion or heterogeneity of the data. The azure line characterizes the IVW prediction, while the deep blue line exhibits the MR-Egger prediction

The MR study did not reveal a cause-and-effect relationship of UC on sarcopenia (IVW: *β* = 0.001, 95% CIs, −0.015–0.017, *p* = 0.576; MR-Egger: *β* = 0.011, 95% CI, −0.029–0.053, *p* = 0.576; weighted median: *β* = 0.014, 95% CIs, −0.010–0.037, *p* = 0.263; simple mode: *β* = 0.010, 95% CIs, −0.035–0.055, *p* = 0.675; weighted mode: *β* = 0.016, 95% CIs, −0.019–0.051, *p* = 0.375) (Fig. 3A, Table 3, in Additional file 1: Fig. S3A). No heterogeneity was detected in certain results (MR-Egger: Q-value = 36.367, *p* = 0.315; IVW: Q-value = 36.713, *p* = 0.344). No potential outliers were detected by MR-PRESSO (*p* = 0.343). The use of the MR-Egger intercept method did not show any substantial indications of directional pleiotropy among the selected IVs (*p* = 0.579), and the funnel plot indicated that the study was not biased (Fig. 3B, Table 3). The leave-one-out examination revealed that none of the individual SNPs had a statistically significant impact on the bias of the overall causal estimates (Additional file 1: Fig. S3B). All selected SNPs included or excluded in the MR analysis for replication are presented in Additional file 2: Table S3.

**Table 3:** MR results for the relationship between UC, CD, and sarcopenia

Method	Number of SNPs	MR analysis			MR-Egger intercept *p*	Heterogeneity *p*
		Beta	95%CI	*p*		
*UC* → *sarcopenia*						
MR Egger	35	0.011	−0.029 to 0.053	0.576	0.579	0.315
Weighted median	35	0.014	−0.010 to 0.037	0.263		
IVW	35	0.001	−0.015 to 0.017	0.901		0.344
Simple mode	35	0.010	−0.035 to 0.055	0.675		
Weighted mode	35	0.016	−0.019 to 0.051	0.375		
*CD* → *sarcopenia*						
MR Egger	50	−0.003	−0.030 to 0.025	0.851	0.668	0.191
Weighted median	50	0.005	−0.014 to 0.024	0.616		
IVW	50	0.003	−0.009 to 0.015	0.656		0.214
Simple mode	50	0.004	−0.031 to 0.038	0.827		
Weighted mode	50	0.005	−0.019 to 0.030	0.672		

*UC* ulcerative colitis, *CD* Crohn’s disease, *FI* frailty index, *IVW* inverse variance weight, *MR* Mendelian randomization

Similarly, the MR results did not uncover a cause-and-effect link between CD and sarcopenia (IVW: *β* = 0.003, 95% CIs, −0.009–0.015, *p* = 0.656; MR-Egger: *β* = −0.003, 95% CIs, −0.030–0.025, *p* = 0.851; weighted median: *β* = 0.005, 95% CIs, −0.014–0.024, *p* = 0.616; simple mode: *β* = 0.004, 95% CIs, −0.031–0.038, *p* = 0.827; weighted mode: *β* = 0.005, 95% CIs, −0.019–0.030, *p* = 0.672) (Fig. 3C, Table 3, in Additional file 1: Fig. S4A). No heterogeneity was detected in certain results (MR-Egger: Q-value = 56.328, *p* = 0.191; IVW: Q-value = 56.547, *p* = 0.214). No potential outliers were detected by MR-PRESSO (*p* = 0.239). Utilizing the MR-Egger intercept approach did not present any significant signs of directional pleiotropy among the chosen IVs (*p* = 0.668), and the funnel plot indicated that the study was not biased (Fig. 3D, Table 3). The leave-one-out valuation demonstrated that none of the individual SNPs had a statistically significant impact on the bias of the overall causal estimates (Additional file 1: Fig. S4B). All selected SNPs included or excluded in the MR analysis for replication are presented in Additional file 2: Table S4.

### Causal effect of FI and sarcopenia on UC and CD

**Fig. 4:**
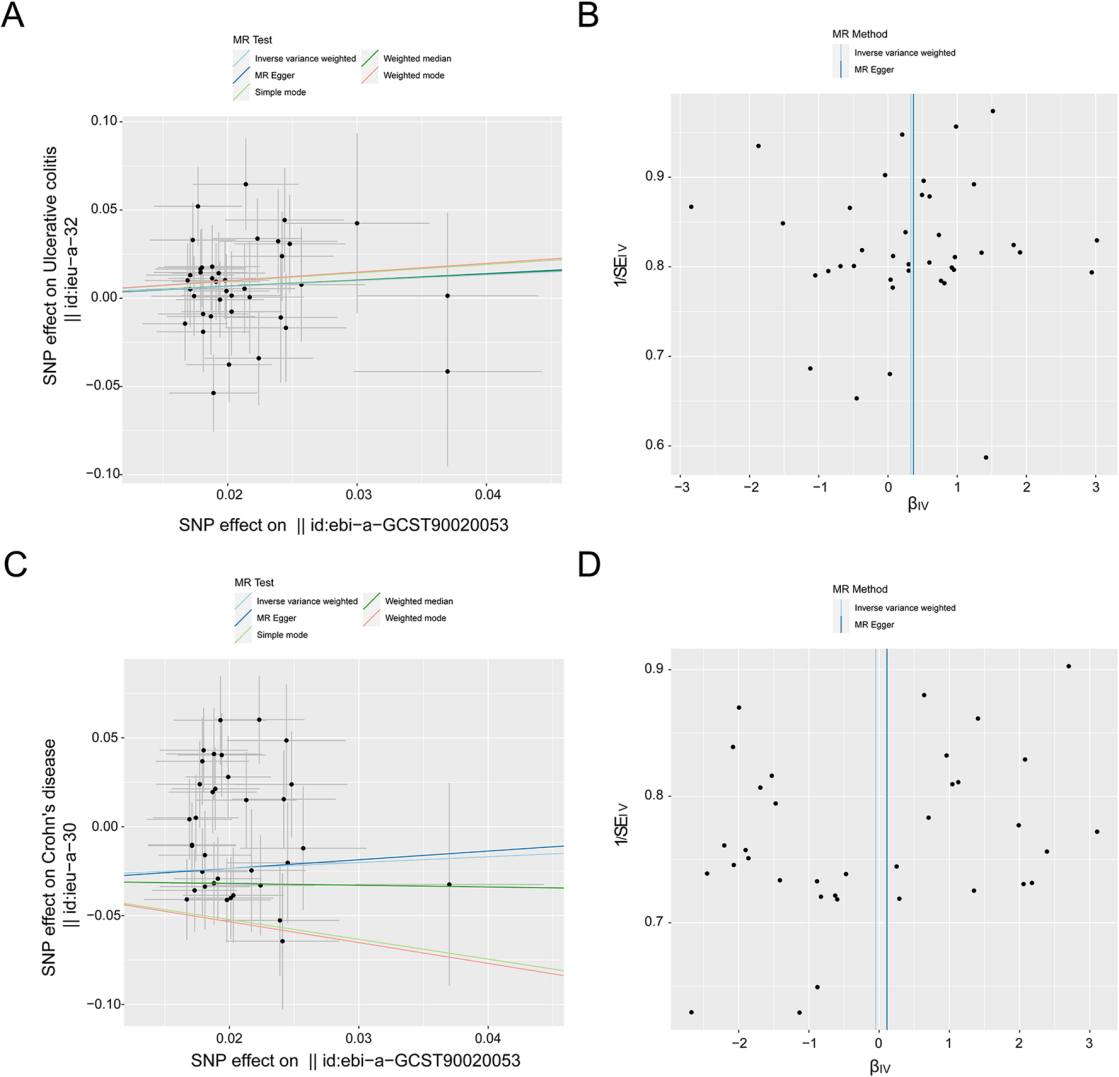
Point diagrams and a funnel illustration were used to portray the causative impact of the frailty index on UC and CD. This encompassed an examination of how the frailty index affects UC (**A**, **B**) and its role in CD (**C**, **D**). The analyses were performed using the standard IVW, simple mode, MR Egger, weighted median, and weighted mode methods. The inclination of each line represents the estimated MR influence per method. A funnel chart was deployed to assess the diversity or dispersion in the data. The azure line characterizes the IVW prediction, while the deep blue line exhibits the MR-Egger prediction

We also conducted a two-sample MR to analyze the cause-and-effect link of FI and sarcopenia on UC and CD. There was no causality between FI and UC (IVW: OR = 1.392, 95% CIs, 0.951–2.038, *p* = 0.089; MR-Egger: OR = 1.446, 95% CIs, 0.123–17.013, *p* = 0.771; weighted median: OR = 1.407, 95% CIs, 0.801–2.473, *p* = 0.235; simple mode: OR = 1.612, 95% CIs, 0.537–4.841, *p* = 0.400; weighted mode: OR = 1.639, 95% CIs, 0.586–4.586, *p* = 0.353) (Fig. 4A, Table 4, in Additional file 1: Fig. S5A). No heterogeneity was detected in some results (MR Egger: Q-value = 37.092, *p* = 0.465; IVW: Q-value = 37.093, *p* = 0.511). No potential outliers were detected by MR-PRESSO (*p* = 0.513). The use of the MR-Egger intercept method did not show any substantial indications of directional pleiotropy among the selected IVs (*p* = 0.976), and the funnel plot indicated that the study was not biased (Fig. 4B, Table 4). The leave-one-out results showed that none of the individual SNPs had a statistically significant impact on the bias of the overall causal estimates (Additional file 1: Fig. S5B). All selected SNPs included or excluded in the MR analysis for replication are presented in Additional file 2: Table S5.

**Table 4:** Bidirectional MR results for the relationship between UC, CD and frailty index

Method	No. of SNPs	MR analysis			MR-Egger intercept *p*	Heterogeneity *p*
		OR	95%CI	*p*		
*FI* → *UC*						
MR Egger	39	1.446	0.123 to 17.013	0.771	0.976	0.465
Weighted median	39	1.407	0.801 to 2.473	0.235		
IVW	39	1.392	0.951 to 2.038	0.089		0.511
Simple mode	39	1.612	0.537 to 4.841	0.400		
Weighted mode	39	1.639	0.586 to 4.586	0.353		
*FI* → *CD*						
MR Egger	35	1.118	0.017 to 74.697	0.959	0.940	0.003
Weighted median	35	0.623	0.316 to 1.228	0.171		
IVW	35	0.952	0.538 to 1.687	0.868		0.004
Simple mode	35	0.226	0.028 to 1.840	0.174		
Weighted mode	35	0.213	0.024 to 1.867	0.172		

*UC* ulcerative colitis, *CD* Crohn’s disease, *FI* frailty index, *IVW* inverse variance weight, *MR* Mendelian randomization, *SNP* single nucleotide polymorphism, *OR* odds ratio, *CI* confidence interval

Similarly, the MR analysis showed that FI had no causality with CD (IVW: OR = 0.952, 95% CIs, 0.538–1.687, *p* = 0.868; MR-Egger: OR = 1.118, 95% CIs, 0.017–74.697, *p* = 0.959; weighted median: OR = 0.623, 95% CIs, 0.316–1.228, *p* = 0.171; simple mode: OR = 0.226, 95% CIs, 0.028–1.840, *p* = 0.174; weighted mode: OR = 0.213, 95% CIs, 0.024–1.867, *p* = 0.172) (Fig. 4C, Table 4, and in Additional file 1: Fig. S6A). Heterogeneity was detected in certain results (MR-Egger: Q-value = 59.715, *p* = 0.003; IVW: Q-value = 59.725, *p* = 0.004). MR-PRESSO detected no potential outliers (*p* = 1.000). The use of the MR-Egger intercept method did not provide any substantial indications of directional pleiotropy among the selected IVs (*p* = 0.940), and the funnel plot showed no bias in the study (Fig. 4D and Table 4). The leave-one-out results showed that none of the individual SNPs had a statistically significant impact on the bias of the overall causal estimates (Additional file 1: Fig. S6B). All the selected SNPs included or excluded in the MR analysis for replication are presented in Additional file 2: Table S6.

**Fig. 5:**
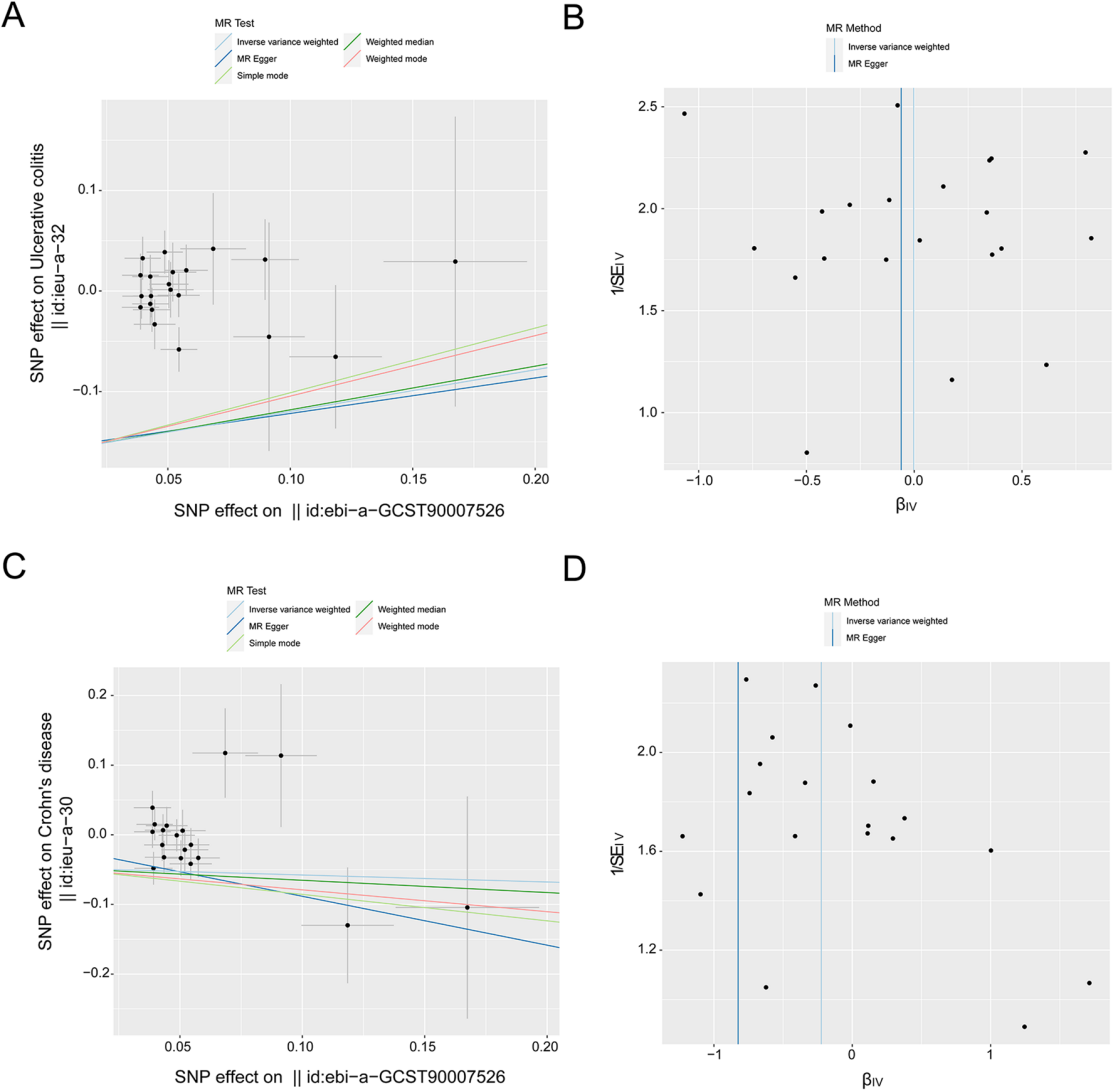
Scatter diagrams and a funnel chart were deployed to depict the cause-and-effect link of sarcopenia on UC and CD. This included an exploration of the impact of sarcopenia on UC (**A**, **B**) and its effect on CD (**C**, **D**). The research was conducted using conventional IVW, simple mode, weighted median, MR Egger, and weighted mode strategies. The gradient of each line symbolizes the approximated MR effect per technique. A funnel chart was selected to gauge the heterogeneity in the data. The azure line characterizes the IVW prediction, while the deep blue line exhibits the MR-Egger prediction

The MR results demonstrated that sarcopenia had a cause–effect link with UC (IVW: OR = 0.998, 95% CIs, 0.796–1.250, *p* = 0.984; MR-Egger: OR = 0.941, 95% CIs, 0.352–2.515, *p* = 0.905; weighted median: OR = 1.017, 95% CIs, 0.730–1.415, *p* = 0.922; simple mode: OR = 1.240, 95% CIs, 0.677–2.273, p = 0.494; weighted mode: OR = 1.192, 95% CIs, 0.692–2.055, *p* = 0.534) (Fig. 5A, Table 5, and in Additional file 1: Fig. S7A). No heterogeneity was detected in certain results (MR-Egger: Q-value = 20.395, *p* = 0.371; IVW: Q-value = 20.410, *p* = 0.433). MR-PRESSO detected no potential outliers (*p* = 0.400). The use of the MR-Egger intercept method did not provide any substantial indications of directional pleiotropy among the selected IVs (*p* = 0.906), and the funnel plot showed no bias in the study (Fig. 5B and Table 5). The leave-one-out results showed that none of the individual SNPs exerted a statistically significant impact on the bias of the overall causal estimates (Additional file 1: Fig. S7B). All the selected SNPs included or excluded in the MR analysis for replication are presented in Additional file 2: Table S7.

**Table 5:** Bidirectional MR results for the relationship between UC, CD, and sarcopenia

Method	No. of SNPs	MR analysis			MR-Egger intercept *p*	Heterogeneity *p*
		OR	95%CI	*p*		
*Sarcopenia* → *UC*						
MR Egger	21	0.941	0.352 to 2.515	0.905	0.906	0.371
Weighted median	21	1.017	0.730 to 1.415	0.922		
IVW	21	0.998	0.796 to 1.250	0.984		0.433
Simple mode	21	1.240	0.677 to 2.273	0.494		
Weighted mode	21	1.192	0.692 to 2.055	0.534		
*Sarcopenia* → *CD*						
MR Egger	19	0.438	0.114 to 1.681	0.245	0.382	0.252
Weighted median	19	0.741	0.515 to 1.067	0.107		
IVW	19	0.799	0.604 to 1.059	0.118		0.259
Simple mode	19	0.605	0.318 to 1.152	0.143		
Weighted mode	19	0.646	0.346 to 1.025	0.186		

*UC* ulcerative colitis, *CD* Crohn’s disease, *FI* frailty index, *IVW* inverse variance weight, *MR* Mendelian randomization, *SNP* single nucleotide polymorphism, *OR* odds ratio, *CI* confidence interval

Similarly, the MR analysis demonstrated that sarcopenia also had no cause-and-effect link with CD (IVW: OR = 0.799, 95% CIs, 0.604–1.059, *p* = 0.118; MR-Egger: OR = 0.438, 95% CIs, 0.114–1.681, *p* = 0.245; weighted median: OR = 0.741, 95% CIs, 0.515–1.067, *p* = 0.107; simple mode: OR = 0.605, 95% CIs, 0.318–2.055, *p* = 0.534; weighted mode: OR = 0.646, 95% CI, 0.346–1.025, *p* = 0.186) (Fig. 5C, Table 5, and in Additional file 1: Fig. S8A). No heterogeneity was detected in certain results (MR-Egger: Q-value = 20.443, *p* = 0.252; IVW: Q-value = 21.410, *p* = 0.259). MR-PRESSO detected no potential outliers (*p* = 0.282). The use of the MR-Egger intercept method did not provide any substantial indications of directional pleiotropy among the selected IVs (*p* = 0.382), and the funnel plot showed no bias in this study (Fig. 5D and Table 5). The leave-one-out results revealed that none of the individual SNPs exerted a statistically significant impact on the bias of the overall causal estimates (Additional file 1: Fig. S8B). All the selected SNPs included or excluded in the MR analysis for replication are presented in Additional file 2: Table S8.

## Discussion

To our understanding, considering the existing research environment, this research signifies an inaugural bidirectional examination utilizing summary-level European data to investigate the causal effects of UC and CD on FI and sarcopenia. Five different estimation methods were used to support the outcomes, and horizontal pleiotropy was addressed and corrected using the MR-PRESSO methodology. Meanwhile, a sensitivity assessment was carried out to confirm the dependability of the causal associations. The findings showed a positive cause-and-effect link between IBD and FI, while no statistically significant causal link was found between IBD and sarcopenia. Conversely, no cause-and-effect link between FI and sarcopenia with UC and CD was observed in this study. These findings indicate a positive association between the occurrence of IBD and a higher incidence of FI. Additionally, no causal relationship was found between IBD and sarcopenia in either cohort examined.

Frailty is a dynamic process that leads to a decline in the function of multiple physiological systems [9]. Multiple frailty assessments including comprehensive geriatric assessment (CGA), Geriatric-8 (G8), and Clinical Frailty Scale (CFS) were commonly performed to identify the risk of frailty in patients [31]. However, the CGA and the G8 were more extensively used by geriatrician for detecting the risk of frailty in all elderly patients [32]. Despite the advantage of these assessments in combining various components in the field of geriatrics, indirect frailty screening methods based on clinical data including the Hospital Frailty Risk Score (HFRS) and the FI were more suitable for exploring the relationship of frailty in large cohorts of populations [33]. The prevalence of frailty in IBD patients ranged from 6% to 39.3% in these published studies using indirect frailty assessments [34]. Although the development of frailty was associated with aging, all IBD patients at every age stage should undergo frailty assessment for early intervention and prevention of adverse outcomes. The FI was constructed by covering a wide range of health domains and could be valuable for research in overall health level. And the FI is a powerful method of estimating frailty, serving as a quantitative variable that can be used as the phenotype of frailty in studies [24]. Previous observational studies have suggested a link between frailty and chronic UC and CD. For instance, a previous study revealed that 6% of the patients were diagnosed with frailty, which was independently associated with increased mortality [8]. Furthermore, another study reported a higher risk of frailty among individuals diagnosed with IBD compared to those without IBD (12% vs 6%) and found that older IBD patients who suffer from frailty were at a greater risk of encountering negative outcomes [35].

IBD progression is associated with systemic, chronic, remitting, and relapsing inflammation, which often results in altered immune function and severe inflammation [36]. Asscher et al. reported that 47.4% elder IBD patients have been diagnosed with frailty, and they found disease activity of IBD was the most significant factor correlated with FI [37]. The inflammatory activity could induce increased risk of muscle atrophy and emotional disorders, all of which could lead to the occurrence of frailty. Meanwhile, malnutrition was secondary to IBD, and this chronic inflammation could trigger tumor necrosis factor (TNF) and chemokines to result in protein-energy malnutrition [38]. Long-term malnutrition, protein loss and chronic inflammation might exacerbate the risk of frailty in patients. Meanwhile, chronic inflammation has been linked to the intrinsic process of aging, including mitochondrial dysfunction, epigenetic alterations, and intrinsic immune cell defects [39]. Consequently, the effects of biological aging are connected to the inflammatory process. Although the precise biological mechanism of frailty is yet to be fully understood, it is widely accepted that frailty is closely associated with chronic inflammation and the aging process [10, 40]. Previous studies hypothesized that the etiology of FI might be linked to the innate immune system, particularly involving signaling pathways related to interferons and chemokines [41]. Additionally, a small cohort study showed that the proinflammatory cytokine, the CXC chemokine ligand-10 (CXCL10) gene, was upregulated in instances of frailty [42]. From these findings, it can be inferred that both UC and CD may influence the development or prevalence of frailty. These findings align with previous research, providing further evidence supporting a potential causal correlation between IBD and frailty. Moreover, a previous study discovered that the frequent recurrence of IBD was associated with younger age [6]. Therefore, frailty could affect younger patients with chronic, relapsing IBD as well, potentially leading to earlier biological aging and influencing clinical outcomes. Kochar BD et al. reported that treating IBD patients with antitumor TNF biologic therapy could improve the progression of frailty [14]. Thus, our results suggested that monitoring frailty in IBD patients and earlier treatment could help to reduce adverse outcomes. Nevertheless, frailty is not causally associated with either UC or CD. While frailty can lead to increased vulnerability to adverse health outcomes, the presence of frailty does not imply a genetic causal association, where frailty causes IBD [8]. Our findings are more likely to show that genetically predicted IBD has a positive causal relationship with frailty rather than frailty leading to IBD. Meanwhile, our findings supported early interventions focusing on disease-related malnutrition and protein loss to resist chronic inflammation and the occurrence of frailty, thereby minimizing the risk of adverse outcomes including persistent muscle atrophy and severe surgical complications in IBD patients.

Our study's results did not reveal any statistically significant causal link between genetically predicted sarcopenia and IBD in either direction. Previous studies demonstrated that approximately 42% of IBD patients were found to have concurrent sarcopenia, which was strongly associated with the clinical outcome of IBD, especially surgical complications [43]. Donnelly M et al. reported that the sarcopenia might be associated with postoperative nutritional impairment and increased morbidity in IBD patients [44]. And Massironi S et al. found that the decrease in muscle mass was associated with delayed wound healing, increased risks of infection, prolonged hospitalization, and increased morbidities as well [45]. The partial mechanisms that drive sarcopenia in IBD patients include inflammation, adiposity, and malabsorption [18]. Proinflammatory cytokines in the bloodstream, such as IFNγ and TNFα, are involved in numerous inflammatory mechanisms and are recognized for their influence on muscle metabolism. Research suggests that elevated levels of IFNγ and TNFα can stimulate muscle protein breakdown (MPB) and suppress muscle protein synthesis (MPS), thereby contributing to the reduction in muscle mass [46]. The adiposity in IBD patients could also produce inflammatory cytokines, which contribute to systemic inflammation and influence muscle mass [47]. Due to intestinal mucosal alterations caused by chronic inflammation in IBD, many patients are in a state of malnutrition, which could exacerbate malabsorption and sarcopenia [48]. Although these mechanisms partially explain the occurrence of sarcopenia in IBD, the causal relationship between IBD and sarcopenia remains unclear. Additionally, sarcopenia is characterized by an age-related decline in muscle mass. Given the relatively young age of participants in the UK Biobank, the sample size for sarcopenia was relatively small in our study. Therefore, the absence of positive results in our study does not mean that the possible causality between IBD and sarcopenia can be entirely ruled out. Future investigations should aim to perform more exhaustive studies with complete data sets and utilize advanced MR techniques to delve deeper into the cause–effect relationship between IBD and sarcopenia. These efforts will contribute to enhancing our understanding of the potential causality between IBD and sarcopenia.

Although the etiologies and disease characteristics have some overlapping and synergistic effects including malnutrition, chronic inflammation, aging and reduced lean mass and physical function, the frailty and the sarcopenia are distinct concepts [49]. In contrast to frailty, the different environmental influencing factors, including metabolic proteins, growth factors, and hormones had an impact on pathophysiology of sarcopenia [50]. Roberts S et al. reviewed that frailty patients could be diagnosed with sarcopenia, but not all sarcopenia patients would have concurrent frailty [51]. Additionally, some studies have already indicated that the pathogenesis of frailty and sarcopenia was influenced in part by genetic factors [52–54]. Therefore, the occurrence of frailty and sarcopenia might be influenced by various factors, including inflammatory disease, aging, and genetic factors. Moreover, frailty and sarcopenia were not completely independent variables, and their mutual influence could result in a lack of significant relationship between IBD and sarcopenia as well.

This study had numerous strengths. First, it was an inaugural study to confirm the causal relationship between IBD, which encompasses UC and CD, and both FI and sarcopenia. This confirmation was achieved through a two-sample bidirectional MR approach. This strategy helped in countering confounding elements, providing a more dependable causal evaluation and elucidation of the direction of causality. Second, to ensure accuracy and validity, we employed five different MR methods. We also utilized MR-PRESSO to acquire consistent estimations of the causal influence, enhancing the robustness of our findings. Third, we explored the causal relationships between IBD and both frailty and sarcopenia, aspects previously overlooked in earlier studies. Last, this study used genetic information as IVs and a large sample size to assess the causal correlation between IBD, FI, and sarcopenia.

However, this study had some limitations. First, we only used the FI phenotype to represent frailty. Multiple assessment methods of frailty have been used in clinical and research settings. Although the indirect frailty screening method including FI was more suitable for exploring the relationship of frailty in large cohorts of populations, FI applicable to overall health might reduce the prevalence of frailty and influence the outcomes. Thus, the assessment method used in this study was limited, which could have affected the reliability of the estimated causality. Second, although this study used summary-level data to estimate causality, certain subgroup analyses, such as age-specific analyses, were limited. However, the incidence of IBD, frailty, and sarcopenia all vary with age. Third, all GWAS in this research were primarily obtained from European individuals. Although this measure could reduce bias from population stratification, it is crucial to mention that the conclusions of the study may not be directly transferrable to other racial or ethnic populations. Last, due to the nature of MR analysis, the estimated causality was derived from the genetic level, providing us with a potential causal relationship rather than a definitive one. However, the specific biological pathway related to this causality should be cautiously determined. The specific biological pathway by which IBD affects the occurrence of frailty and sarcopenia needs to be further explored.

In conclusion, this study provided solid evidence that UC and CD had a causal and positive correlation with FI; in other words, the occurrence of IBD might be a possible predictor of frailty and IBD patients might exhibit aging-related characteristics. This study suggests that frailty should be assessed as early as possible in IBD patients, and preventive measures for frailty should be taken to reduce adverse IBD outcomes.

### Supplementary Information

**Additional file 1: Figure S1.** Causal effect and sensitivity analysis of UC-associated single nucleotide polymorphisms on FI. (A) A forest chart illustrating the causative impacts of years of UC-related SNPs on the FI. (B) A sensitivity analysis was performed to explore the likelihood that the causal link was propelled by a distinctive SNP. **Figure S2.** Investigation into the causative role and sensitivity analysis of CD-associated SNPs on FI. (A) Forest plot depicting the causal effects of the duration of CD-linked single nucleotide polymorphisms on FI. (B) A detailed sensitivity review was carried out to explore the possibility that a unique SNP was the driving factor behind the causal association. **Figure S3.** Investigation into the causative role and sensitivity analysis of UC-linked SNPs on sarcopenia. (A) Forest plot illustrating the causal implications of the duration of UC-related SNPs on sarcopenia. (B) A detailed sensitivity review was conducted to assess the potential that a unique SNP was the driving force behind the causal association. **Figure S4.** Investigation into the causative influence and sensitivity examination of CD-related SNPs on sarcopenia. (A) Forest plot displaying the causal effects of the duration of CD-related SNPs on sarcopenia. (B) A detailed sensitivity review was carried out to evaluate the probability that a distinct SNP was the driving factor behind the causal association. **Figure S5.** Analysis of the causative role and sensitivity assessment of FI-linked SNPs in UC. (A) Forest plot depicting the causal impacts of the duration of FI-related SNPs on UC. (B) A sensitivity examination conducted to delve into the likelihood that the causal link was driven by a distinctive SNP. **Figure S6.** Analysis of the causative role and sensitivity study of FI-related SNPs in CD. (A) Forest plot demonstrating the causal effects of the duration of FI-linked single nucleotide polymorphisms on CD. (B) A detailed sensitivity review was carried out to assess the possibility that a unique SNP was the driving factor behind the causal association. **Figure S7.** Investigation into the causative influence and sensitivity assessment of sarcopenia-linked SNPs on UC. (A) Forest plot illustrating the causal implications of years of sarcopenia-associated SNPs on UC. (B) A sensitivity analysis conducted to probe into the likelihood that the causal connection was instigated by a distinct SNP. **Figure S8.** Investigation into the causative influence and sensitivity assessment of sarcopenia-linked SNPs on CD. (A) Forest plot illustrating the causal implications of years of sarcopenia-associated SNPs on CD. (B) A sensitivity analysis conducted to probe into the likelihood that the causal connection was instigated by a distinct SNP.

**Additional file 2: Table S1.** Characteristics of SNPs included in the causative relationship of UC with frailty index. **Table S2.** Characteristics of SNPs included in the causative relationship of CD with frailty index. **Table S3.** Characteristics of SNPs included in the causative relationship of UC with sarcopenia. **Table S4.** Characteristics of SNPs included in the causative relationship of CD with sarcopenia. **Table S5.** Characteristics of SNPs included in the causative relationship of frailty index with UC. **Table S6.** Characteristics of SNPs included in the causative relationship of frailty index with CD. **Table S7.** Characteristics of SNPs included in the causative relationship of sarcopenia with UC. **Table S8.** Characteristics of SNPs included in the causative relationship of sarcopenia with CD.

## Data Availability

The datasets supporting the conclusion of the article are included within the article and additional files, and the data generated or analyzed during this study are available in this published article and its supplementary information files.

## Abbreviations

MR: Mendelian randomization
IBD: Inflammatory bowel disease
GWAS: Genome-wide association studies
FI: Frailty index
IVW: Inverse variance weight
UC: Ulcerative colitis
CD: Crohn's disease
IV: Instrumental variables
SNP: Single nucleotide polymorphism
MR-PRESSO: Mendelian randomization pleiotropy residual sum and outlier
CXCL10: CXC chemokine ligand-10
TNF: Tumor necrosis factor
MPB: Muscle protein breakdown
MPS: Muscle protein synthesis
