# Supplementary figures and images for: Assessing causality between inflammatory bowel diseases with frailty index and sarcopenia: a bidirectional Mendelian randomization study
Source: Eur J Med Res. 2024 Jan 5;29:23. doi: 10.1186/s40001-023-01614-5 (PMC10768401; doi:10.1186/s40001-023-01614-5)

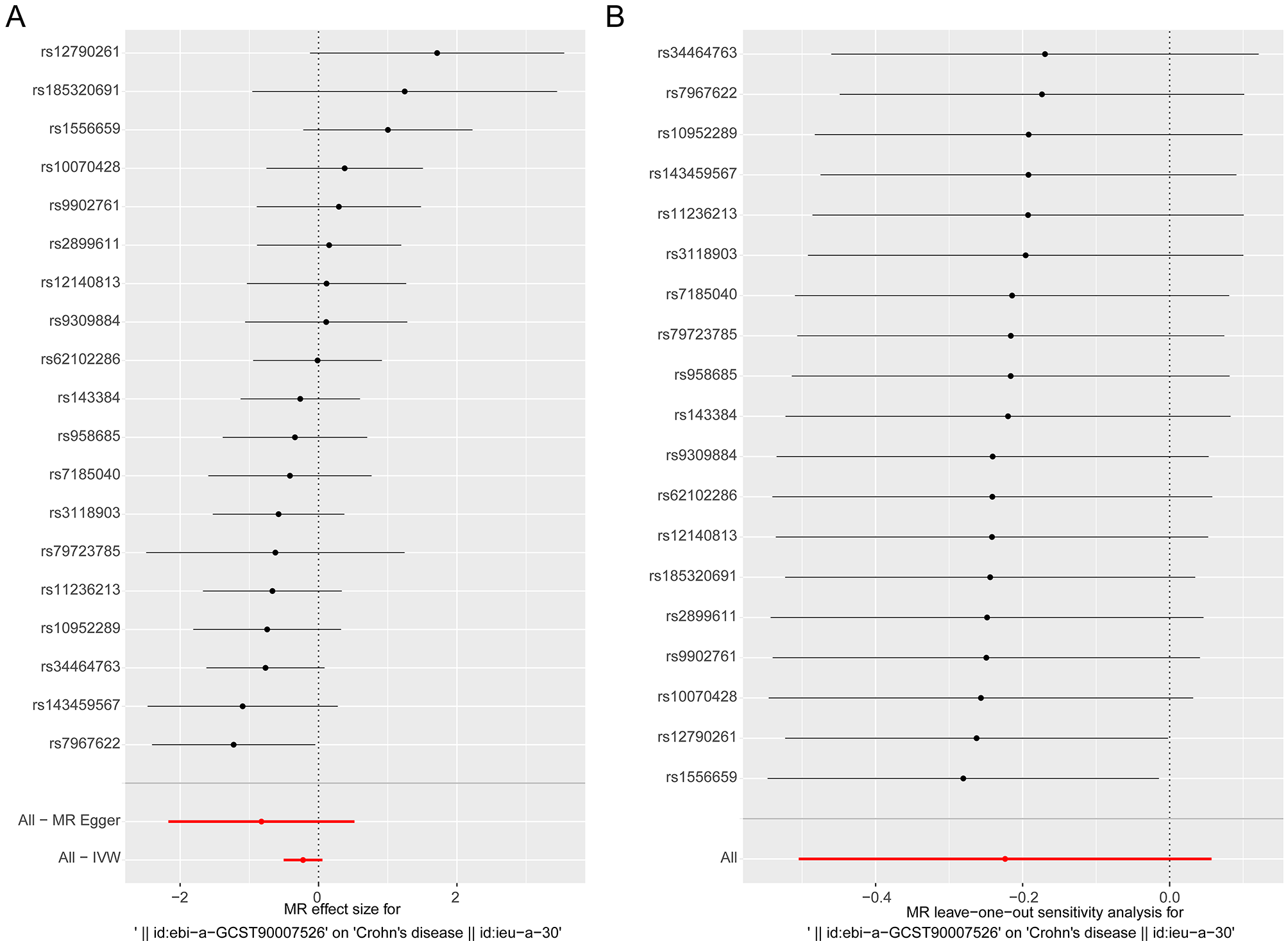

Supplement: Supplementary file 1 — Additional file 1: Figure S1. Causal effect and sensitivity analysis of UC-associated single nucleotide polymorphisms on FI. (A) A forest chart illustrating the causative impacts of years of UC-related SNPs on the FI. (B) A sensitivity analysis was performed to explore the likelihood that the causal link was propelled by a distinctive SNP. Figure S2. Investigation into the causative role and sensitivity analysis of CD-associated SNPs on FI. (A) Forest plot depicting the causal effects of the duration of CD-linked single nucleotide polymorphisms on FI. (B) A detailed sensitivity review was carried out to explore the possibility that a unique SNP was the driving factor behind the causal association. Figure S3. Investigation into the causative role and sensitivity analysis of UC-linked SNPs on sarcopenia. (A) Forest plot illustrating the causal implications of the duration of UC-related SNPs on sarcopenia. (B) A detailed sensitivity review was conducted to assess the potential that a unique SNP was the driving force behind the causal association. Figure S4. Investigation into the causative influence and sensitivity examination of CD-related SNPs on sarcopenia. (A) Forest plot displaying the causal effects of the duration of CD-related SNPs on sarcopenia. (B) A detailed sensitivity review was carried out to evaluate the probability that a distinct SNP was the driving factor behind the causal association. Figure S5. Analysis of the causative role and sensitivity assessment of FI-linked SNPs in UC. (A) Forest plot depicting the causal impacts of the duration of FI-related SNPs on UC. (B) A sensitivity examination conducted to delve into the likelihood that the causal link was driven by a distinctive SNP. Figure S6. Analysis of the causative role and sensitivity study of FI-related SNPs in CD. (A) Forest plot demonstrating the causal effects of the duration of FI-linked single nucleotide polymorphisms on CD. (B) A detailed sensitivity review was carried out to assess the p [file 40001_2023_1614_MOESM1_ESM.zip › Additional file 1/Supplement Figure 8.tif]

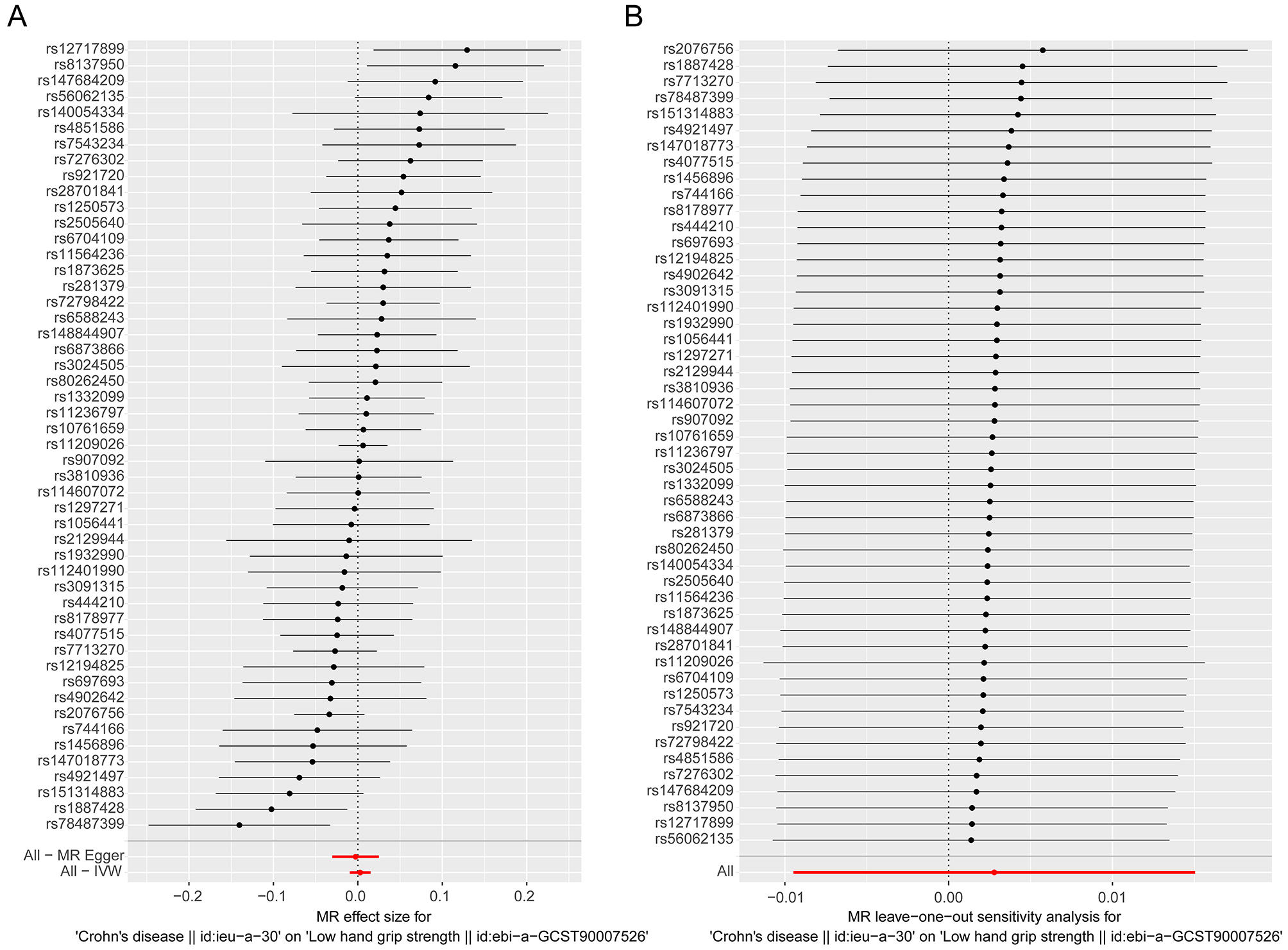

Supplement: Supplementary file 1 — Additional file 1: Figure S1. Causal effect and sensitivity analysis of UC-associated single nucleotide polymorphisms on FI. (A) A forest chart illustrating the causative impacts of years of UC-related SNPs on the FI. (B) A sensitivity analysis was performed to explore the likelihood that the causal link was propelled by a distinctive SNP. Figure S2. Investigation into the causative role and sensitivity analysis of CD-associated SNPs on FI. (A) Forest plot depicting the causal effects of the duration of CD-linked single nucleotide polymorphisms on FI. (B) A detailed sensitivity review was carried out to explore the possibility that a unique SNP was the driving factor behind the causal association. Figure S3. Investigation into the causative role and sensitivity analysis of UC-linked SNPs on sarcopenia. (A) Forest plot illustrating the causal implications of the duration of UC-related SNPs on sarcopenia. (B) A detailed sensitivity review was conducted to assess the potential that a unique SNP was the driving force behind the causal association. Figure S4. Investigation into the causative influence and sensitivity examination of CD-related SNPs on sarcopenia. (A) Forest plot displaying the causal effects of the duration of CD-related SNPs on sarcopenia. (B) A detailed sensitivity review was carried out to evaluate the probability that a distinct SNP was the driving factor behind the causal association. Figure S5. Analysis of the causative role and sensitivity assessment of FI-linked SNPs in UC. (A) Forest plot depicting the causal impacts of the duration of FI-related SNPs on UC. (B) A sensitivity examination conducted to delve into the likelihood that the causal link was driven by a distinctive SNP. Figure S6. Analysis of the causative role and sensitivity study of FI-related SNPs in CD. (A) Forest plot demonstrating the causal effects of the duration of FI-linked single nucleotide polymorphisms on CD. (B) A detailed sensitivity review was carried out to assess the p [file 40001_2023_1614_MOESM1_ESM.zip › Additional file 1/Supplement Figure 4.tif]

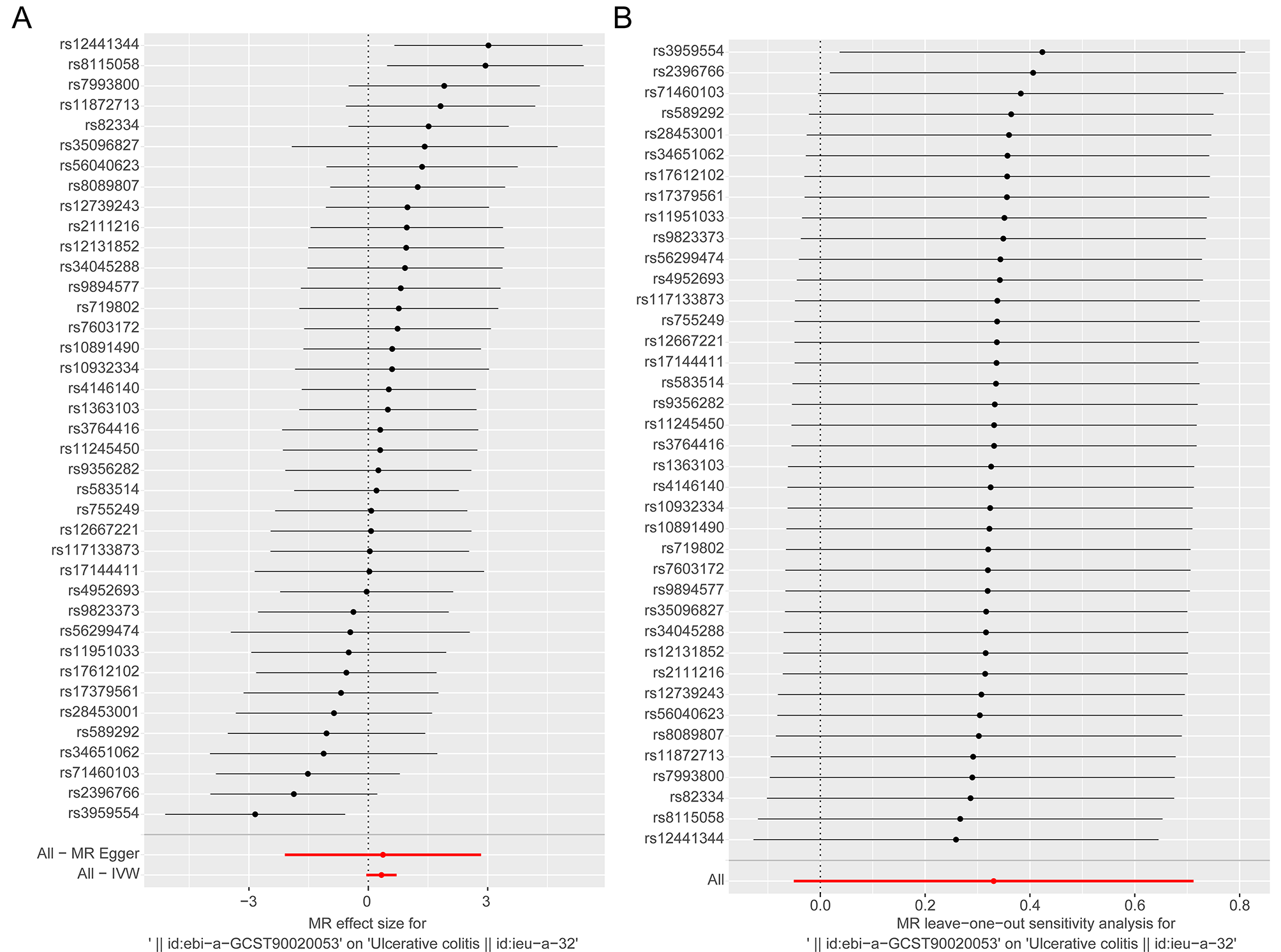

Supplement: Supplementary file 1 — Additional file 1: Figure S1. Causal effect and sensitivity analysis of UC-associated single nucleotide polymorphisms on FI. (A) A forest chart illustrating the causative impacts of years of UC-related SNPs on the FI. (B) A sensitivity analysis was performed to explore the likelihood that the causal link was propelled by a distinctive SNP. Figure S2. Investigation into the causative role and sensitivity analysis of CD-associated SNPs on FI. (A) Forest plot depicting the causal effects of the duration of CD-linked single nucleotide polymorphisms on FI. (B) A detailed sensitivity review was carried out to explore the possibility that a unique SNP was the driving factor behind the causal association. Figure S3. Investigation into the causative role and sensitivity analysis of UC-linked SNPs on sarcopenia. (A) Forest plot illustrating the causal implications of the duration of UC-related SNPs on sarcopenia. (B) A detailed sensitivity review was conducted to assess the potential that a unique SNP was the driving force behind the causal association. Figure S4. Investigation into the causative influence and sensitivity examination of CD-related SNPs on sarcopenia. (A) Forest plot displaying the causal effects of the duration of CD-related SNPs on sarcopenia. (B) A detailed sensitivity review was carried out to evaluate the probability that a distinct SNP was the driving factor behind the causal association. Figure S5. Analysis of the causative role and sensitivity assessment of FI-linked SNPs in UC. (A) Forest plot depicting the causal impacts of the duration of FI-related SNPs on UC. (B) A sensitivity examination conducted to delve into the likelihood that the causal link was driven by a distinctive SNP. Figure S6. Analysis of the causative role and sensitivity study of FI-related SNPs in CD. (A) Forest plot demonstrating the causal effects of the duration of FI-linked single nucleotide polymorphisms on CD. (B) A detailed sensitivity review was carried out to assess the p [file 40001_2023_1614_MOESM1_ESM.zip › Additional file 1/Supplement Figure 5.tif]

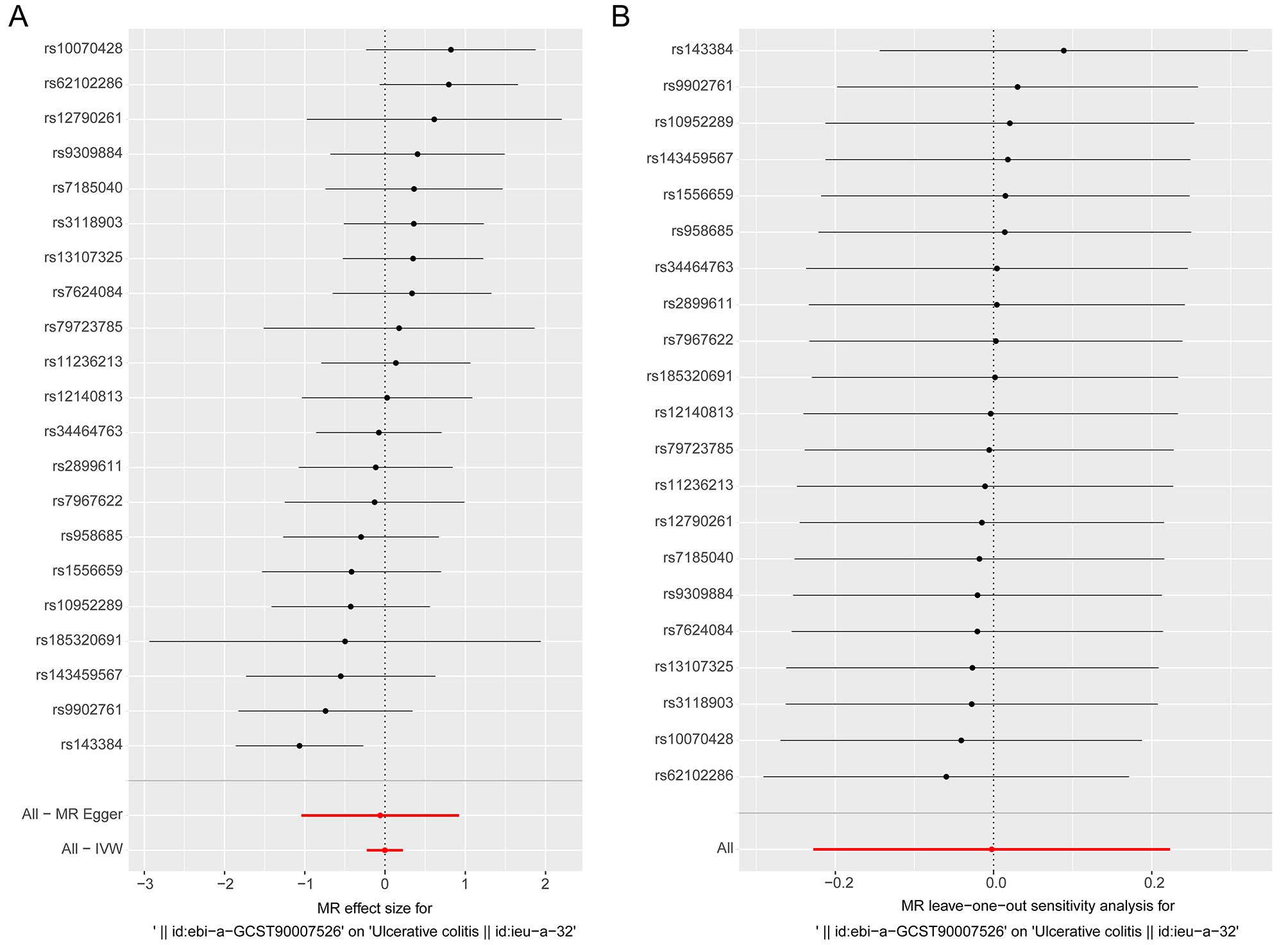

Supplement: Supplementary file 1 — Additional file 1: Figure S1. Causal effect and sensitivity analysis of UC-associated single nucleotide polymorphisms on FI. (A) A forest chart illustrating the causative impacts of years of UC-related SNPs on the FI. (B) A sensitivity analysis was performed to explore the likelihood that the causal link was propelled by a distinctive SNP. Figure S2. Investigation into the causative role and sensitivity analysis of CD-associated SNPs on FI. (A) Forest plot depicting the causal effects of the duration of CD-linked single nucleotide polymorphisms on FI. (B) A detailed sensitivity review was carried out to explore the possibility that a unique SNP was the driving factor behind the causal association. Figure S3. Investigation into the causative role and sensitivity analysis of UC-linked SNPs on sarcopenia. (A) Forest plot illustrating the causal implications of the duration of UC-related SNPs on sarcopenia. (B) A detailed sensitivity review was conducted to assess the potential that a unique SNP was the driving force behind the causal association. Figure S4. Investigation into the causative influence and sensitivity examination of CD-related SNPs on sarcopenia. (A) Forest plot displaying the causal effects of the duration of CD-related SNPs on sarcopenia. (B) A detailed sensitivity review was carried out to evaluate the probability that a distinct SNP was the driving factor behind the causal association. Figure S5. Analysis of the causative role and sensitivity assessment of FI-linked SNPs in UC. (A) Forest plot depicting the causal impacts of the duration of FI-related SNPs on UC. (B) A sensitivity examination conducted to delve into the likelihood that the causal link was driven by a distinctive SNP. Figure S6. Analysis of the causative role and sensitivity study of FI-related SNPs in CD. (A) Forest plot demonstrating the causal effects of the duration of FI-linked single nucleotide polymorphisms on CD. (B) A detailed sensitivity review was carried out to assess the p [file 40001_2023_1614_MOESM1_ESM.zip › Additional file 1/Supplement Figure 7.tif]

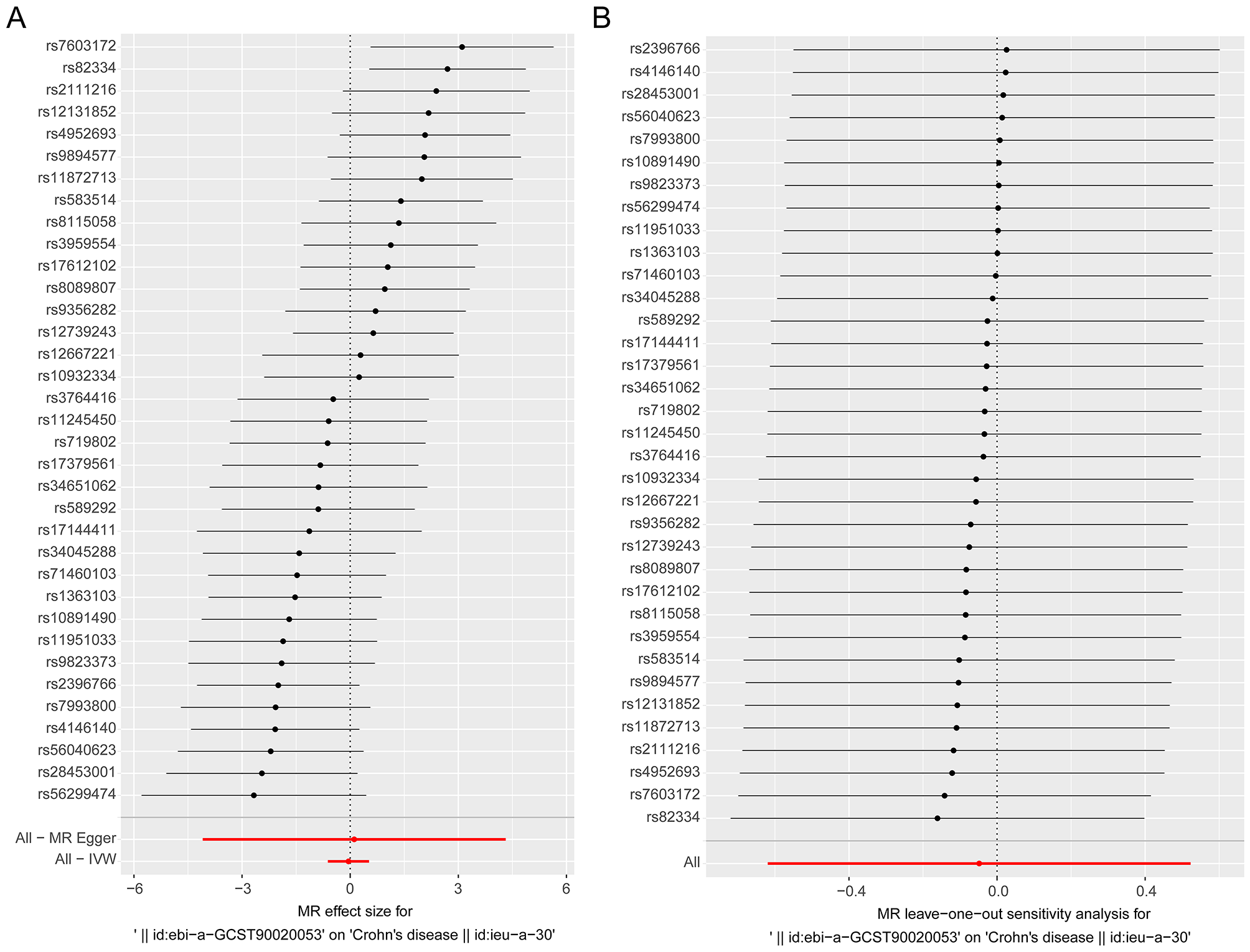

Supplement: Supplementary file 1 — Additional file 1: Figure S1. Causal effect and sensitivity analysis of UC-associated single nucleotide polymorphisms on FI. (A) A forest chart illustrating the causative impacts of years of UC-related SNPs on the FI. (B) A sensitivity analysis was performed to explore the likelihood that the causal link was propelled by a distinctive SNP. Figure S2. Investigation into the causative role and sensitivity analysis of CD-associated SNPs on FI. (A) Forest plot depicting the causal effects of the duration of CD-linked single nucleotide polymorphisms on FI. (B) A detailed sensitivity review was carried out to explore the possibility that a unique SNP was the driving factor behind the causal association. Figure S3. Investigation into the causative role and sensitivity analysis of UC-linked SNPs on sarcopenia. (A) Forest plot illustrating the causal implications of the duration of UC-related SNPs on sarcopenia. (B) A detailed sensitivity review was conducted to assess the potential that a unique SNP was the driving force behind the causal association. Figure S4. Investigation into the causative influence and sensitivity examination of CD-related SNPs on sarcopenia. (A) Forest plot displaying the causal effects of the duration of CD-related SNPs on sarcopenia. (B) A detailed sensitivity review was carried out to evaluate the probability that a distinct SNP was the driving factor behind the causal association. Figure S5. Analysis of the causative role and sensitivity assessment of FI-linked SNPs in UC. (A) Forest plot depicting the causal impacts of the duration of FI-related SNPs on UC. (B) A sensitivity examination conducted to delve into the likelihood that the causal link was driven by a distinctive SNP. Figure S6. Analysis of the causative role and sensitivity study of FI-related SNPs in CD. (A) Forest plot demonstrating the causal effects of the duration of FI-linked single nucleotide polymorphisms on CD. (B) A detailed sensitivity review was carried out to assess the p [file 40001_2023_1614_MOESM1_ESM.zip › Additional file 1/Supplement Figure 6.tif]

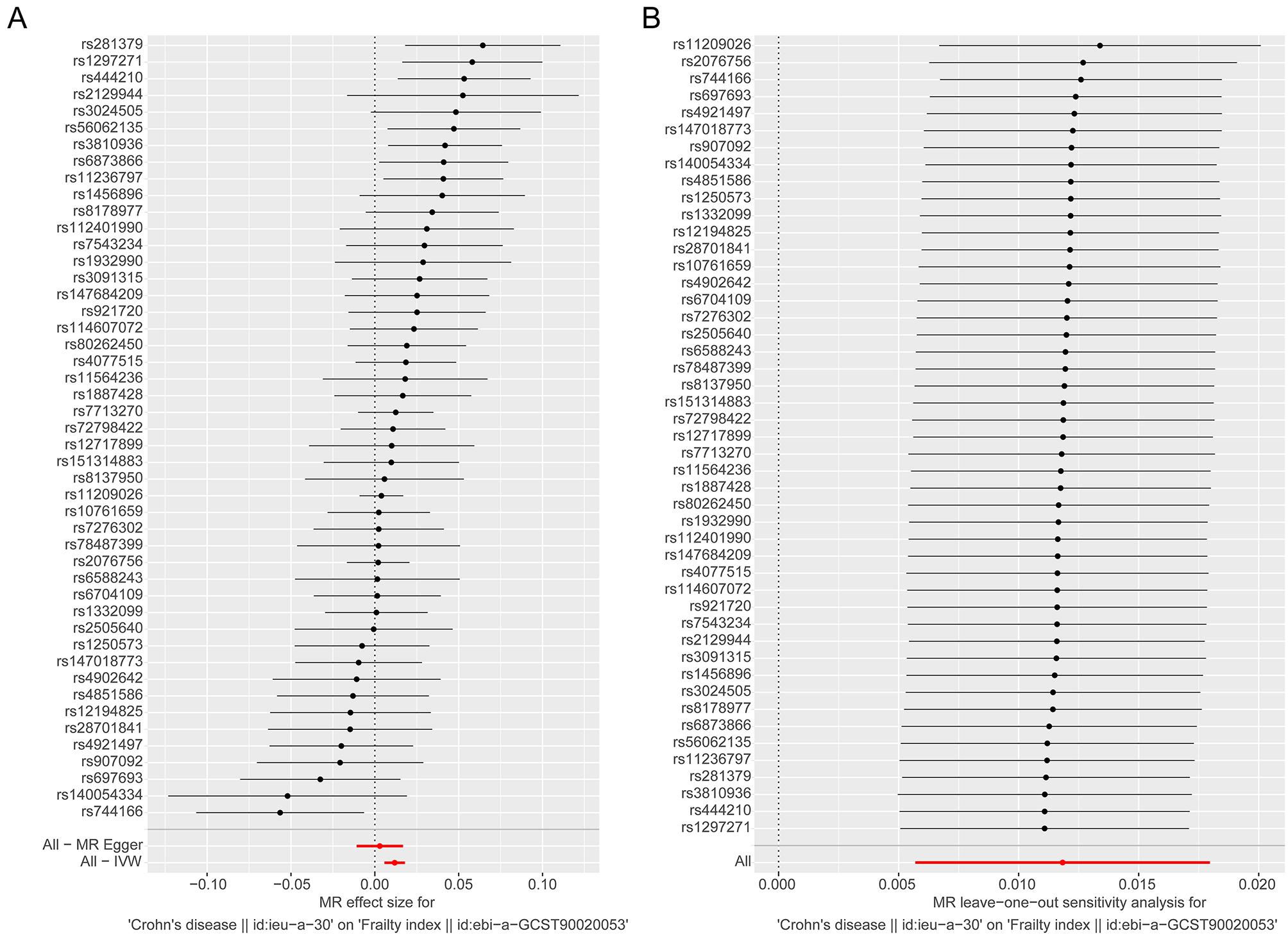

Supplement: Supplementary file 1 — Additional file 1: Figure S1. Causal effect and sensitivity analysis of UC-associated single nucleotide polymorphisms on FI. (A) A forest chart illustrating the causative impacts of years of UC-related SNPs on the FI. (B) A sensitivity analysis was performed to explore the likelihood that the causal link was propelled by a distinctive SNP. Figure S2. Investigation into the causative role and sensitivity analysis of CD-associated SNPs on FI. (A) Forest plot depicting the causal effects of the duration of CD-linked single nucleotide polymorphisms on FI. (B) A detailed sensitivity review was carried out to explore the possibility that a unique SNP was the driving factor behind the causal association. Figure S3. Investigation into the causative role and sensitivity analysis of UC-linked SNPs on sarcopenia. (A) Forest plot illustrating the causal implications of the duration of UC-related SNPs on sarcopenia. (B) A detailed sensitivity review was conducted to assess the potential that a unique SNP was the driving force behind the causal association. Figure S4. Investigation into the causative influence and sensitivity examination of CD-related SNPs on sarcopenia. (A) Forest plot displaying the causal effects of the duration of CD-related SNPs on sarcopenia. (B) A detailed sensitivity review was carried out to evaluate the probability that a distinct SNP was the driving factor behind the causal association. Figure S5. Analysis of the causative role and sensitivity assessment of FI-linked SNPs in UC. (A) Forest plot depicting the causal impacts of the duration of FI-related SNPs on UC. (B) A sensitivity examination conducted to delve into the likelihood that the causal link was driven by a distinctive SNP. Figure S6. Analysis of the causative role and sensitivity study of FI-related SNPs in CD. (A) Forest plot demonstrating the causal effects of the duration of FI-linked single nucleotide polymorphisms on CD. (B) A detailed sensitivity review was carried out to assess the p [file 40001_2023_1614_MOESM1_ESM.zip › Additional file 1/Supplement Figure 2.tif]

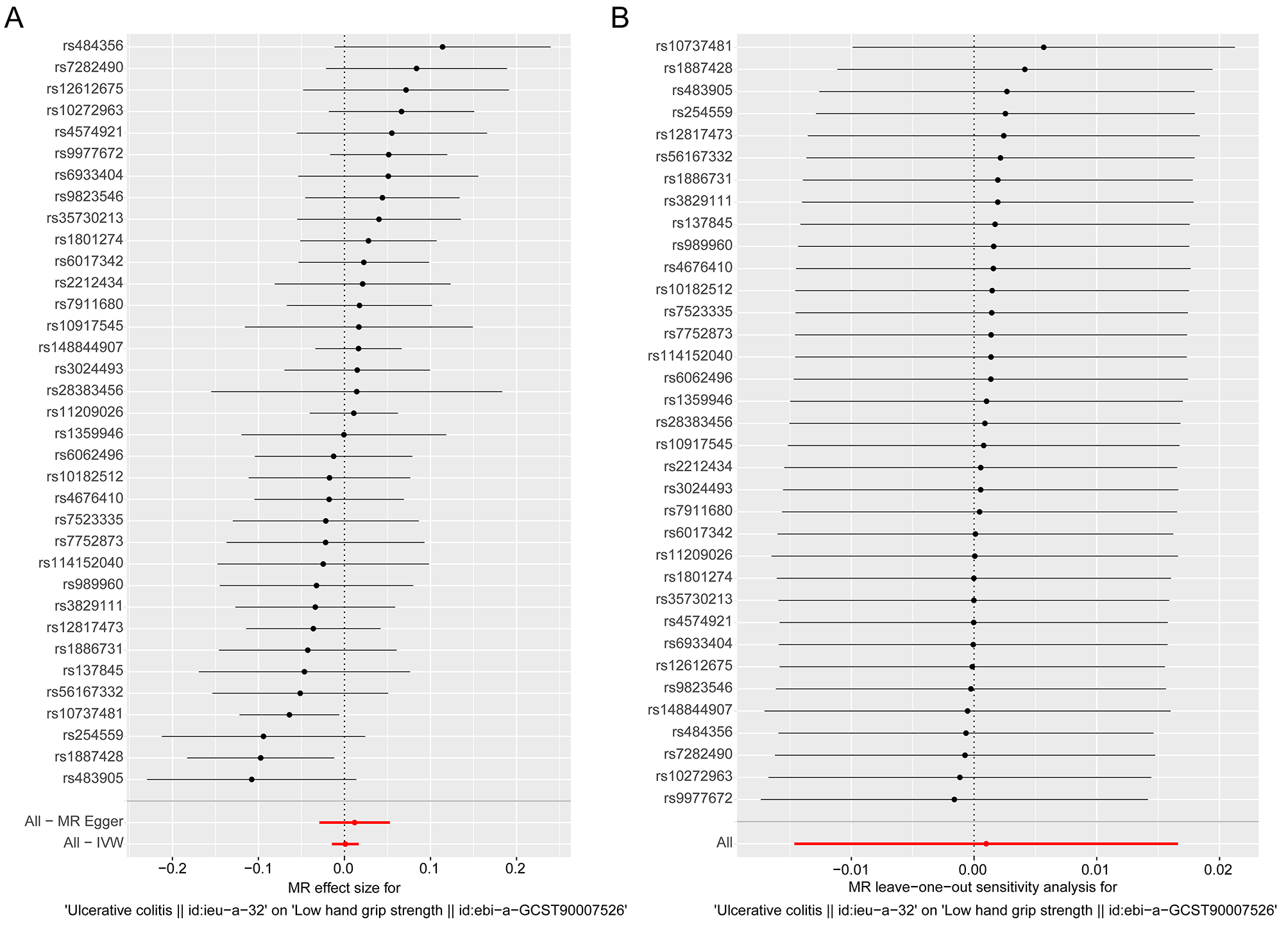

Supplement: Supplementary file 1 — Additional file 1: Figure S1. Causal effect and sensitivity analysis of UC-associated single nucleotide polymorphisms on FI. (A) A forest chart illustrating the causative impacts of years of UC-related SNPs on the FI. (B) A sensitivity analysis was performed to explore the likelihood that the causal link was propelled by a distinctive SNP. Figure S2. Investigation into the causative role and sensitivity analysis of CD-associated SNPs on FI. (A) Forest plot depicting the causal effects of the duration of CD-linked single nucleotide polymorphisms on FI. (B) A detailed sensitivity review was carried out to explore the possibility that a unique SNP was the driving factor behind the causal association. Figure S3. Investigation into the causative role and sensitivity analysis of UC-linked SNPs on sarcopenia. (A) Forest plot illustrating the causal implications of the duration of UC-related SNPs on sarcopenia. (B) A detailed sensitivity review was conducted to assess the potential that a unique SNP was the driving force behind the causal association. Figure S4. Investigation into the causative influence and sensitivity examination of CD-related SNPs on sarcopenia. (A) Forest plot displaying the causal effects of the duration of CD-related SNPs on sarcopenia. (B) A detailed sensitivity review was carried out to evaluate the probability that a distinct SNP was the driving factor behind the causal association. Figure S5. Analysis of the causative role and sensitivity assessment of FI-linked SNPs in UC. (A) Forest plot depicting the causal impacts of the duration of FI-related SNPs on UC. (B) A sensitivity examination conducted to delve into the likelihood that the causal link was driven by a distinctive SNP. Figure S6. Analysis of the causative role and sensitivity study of FI-related SNPs in CD. (A) Forest plot demonstrating the causal effects of the duration of FI-linked single nucleotide polymorphisms on CD. (B) A detailed sensitivity review was carried out to assess the p [file 40001_2023_1614_MOESM1_ESM.zip › Additional file 1/Supplement Figure 3.tif]

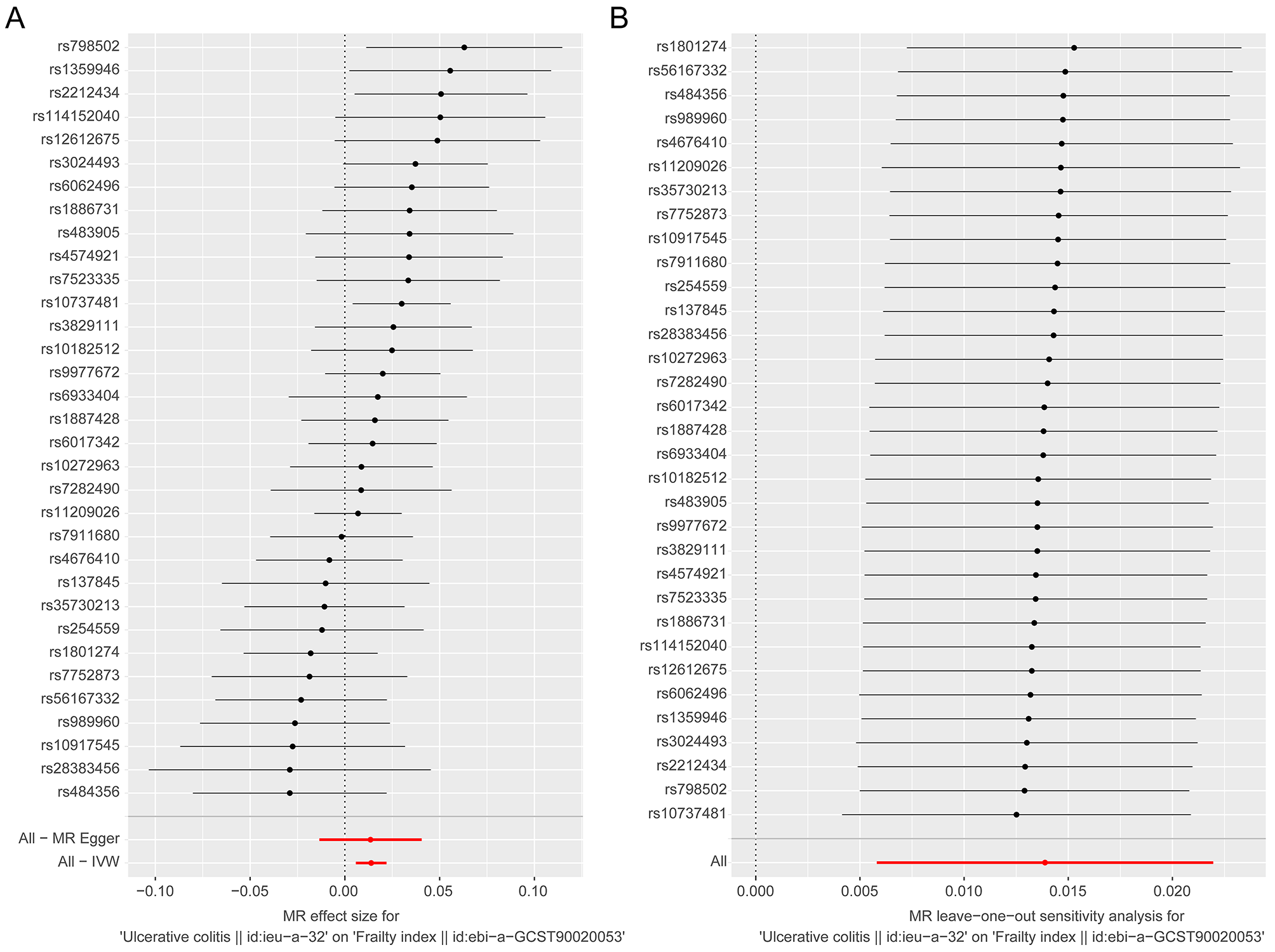

Supplement: Supplementary file 1 — Additional file 1: Figure S1. Causal effect and sensitivity analysis of UC-associated single nucleotide polymorphisms on FI. (A) A forest chart illustrating the causative impacts of years of UC-related SNPs on the FI. (B) A sensitivity analysis was performed to explore the likelihood that the causal link was propelled by a distinctive SNP. Figure S2. Investigation into the causative role and sensitivity analysis of CD-associated SNPs on FI. (A) Forest plot depicting the causal effects of the duration of CD-linked single nucleotide polymorphisms on FI. (B) A detailed sensitivity review was carried out to explore the possibility that a unique SNP was the driving factor behind the causal association. Figure S3. Investigation into the causative role and sensitivity analysis of UC-linked SNPs on sarcopenia. (A) Forest plot illustrating the causal implications of the duration of UC-related SNPs on sarcopenia. (B) A detailed sensitivity review was conducted to assess the potential that a unique SNP was the driving force behind the causal association. Figure S4. Investigation into the causative influence and sensitivity examination of CD-related SNPs on sarcopenia. (A) Forest plot displaying the causal effects of the duration of CD-related SNPs on sarcopenia. (B) A detailed sensitivity review was carried out to evaluate the probability that a distinct SNP was the driving factor behind the causal association. Figure S5. Analysis of the causative role and sensitivity assessment of FI-linked SNPs in UC. (A) Forest plot depicting the causal impacts of the duration of FI-related SNPs on UC. (B) A sensitivity examination conducted to delve into the likelihood that the causal link was driven by a distinctive SNP. Figure S6. Analysis of the causative role and sensitivity study of FI-related SNPs in CD. (A) Forest plot demonstrating the causal effects of the duration of FI-linked single nucleotide polymorphisms on CD. (B) A detailed sensitivity review was carried out to assess the p [file 40001_2023_1614_MOESM1_ESM.zip › Additional file 1/Supplement Figure 1.tif]
